# Combined Associations of a Polygenic Risk Score and Classical Risk Factors With Breast Cancer Risk

**DOI:** 10.1093/jnci/djaa056

**Published:** 2020-05-02

**Authors:** Pooja Middha Kapoor, Nasim Mavaddat, Parichoy Pal Choudhury, Amber N Wilcox, Sara Lindström, Sabine Behrens, Kyriaki Michailidou, Joe Dennis, Manjeet K Bolla, Qin Wang, Audrey Jung, Zomoroda Abu-Ful, Thomas Ahearn, Irene L Andrulis, Hoda Anton-Culver, Volker Arndt, Kristan J Aronson, Paul L Auer, Laura E Beane Freeman, Heiko Becher, Matthias W Beckmann, Alicia Beeghly-Fadiel, Javier Benitez, Leslie Bernstein, Stig E Bojesen, Hiltrud Brauch, Hermann Brenner, Thomas Brüning, Qiuyin Cai, Daniele Campa, Federico Canzian, Angel Carracedo, Brian D Carter, Jose E Castelao, Stephen J Chanock, Nilanjan Chatterjee, Georgia Chenevix-Trench, Christine L Clarke, Fergus J Couch, Angela Cox, Simon S Cross, Kamila Czene, James Y Dai, H Shelton Earp, Arif B Ekici, A Heather Eliassen, Mikael Eriksson, D Gareth Evans, Peter A Fasching, Jonine Figueroa, Lin Fritschi, Marike Gabrielson, Manuela Gago-Dominguez, Chi Gao, Susan M Gapstur, Mia M Gaudet, Graham G Giles, Anna González-Neira, Pascal Guénel, Lothar Haeberle, Christopher A Haiman, Niclas Håkansson, Per Hall, Ute Hamann, Sigrid Hatse, Jane Heyworth, Bernd Holleczek, Robert N Hoover, John L Hopper, Anthony Howell, David J Hunter, ABCTB Investigators, kConFab/AOCS Investigators, Esther M John, Michael E Jones, Rudolf Kaaks, Renske Keeman, Cari M Kitahara, Yon-Dschun Ko, Stella Koutros, Allison W Kurian, Diether Lambrechts, Loic Le Marchand, Eunjung Lee, Flavio Lejbkowicz, Martha Linet, Jolanta Lissowska, Ana Llaneza, Robert J MacInnis, Maria Elena Martinez, Tabea Maurer, Catriona McLean, Susan L Neuhausen, William G Newman, Aaron Norman, Katie M O’Brien, Andrew F Olshan, Janet E Olson, Håkan Olsson, Nick Orr, Charles M Perou, Guillermo Pita, Eric C Polley, Ross L Prentice, Gad Rennert, Hedy S Rennert, Kathryn J Ruddy, Dale P Sandler, Christobel Saunders, Minouk J Schoemaker, Ben Schöttker, Fredrick Schumacher, Christopher Scott, Rodney J Scott, Xiao-Ou Shu, Ann Smeets, Melissa C Southey, John J Spinelli, Jennifer Stone, Anthony J Swerdlow, Rulla M Tamimi, Jack A Taylor, Melissa A Troester, Celine M Vachon, Elke M van Veen, Xiaoliang Wang, Clarice R Weinberg, Caroline Weltens, Walter Willett, Stacey J Winham, Alicja Wolk, Xiaohong R Yang, Wei Zheng, Argyrios Ziogas, Alison M Dunning, Paul D P Pharoah, Marjanka K Schmidt, Peter Kraft, Douglas F Easton, Roger L Milne, Montserrat García-Closas, Jenny Chang-Claude

**Affiliations:** 1 Division of Cancer Epidemiology, German Cancer Research Center (DKFZ), Heidelberg, Germany; 2 Faculty of Medicine, University of Heidelberg, Heidelberg, Germany; 3 Centre for Cancer Genetic Epidemiology, Department of Public Health and Primary Care, University of Cambridge, Cambridge, UK; 4 Division of Cancer Epidemiology and Genetics, Department of Health and Human Services, National Cancer Institute, National Institutes of Health, Bethesda, MD, USA; 5 Department of Epidemiology, Gillings School of Global Public Health, University of North Carolina at Chapel Hill, Chapel Hill, NC, USA; 6 Department of Epidemiology, University of Washington School of Public Health, Seattle, WA, USA; 7 Public Health Sciences Division, Fred Hutchinson Cancer Research Center, Seattle, WA, USA; 8 Biostatistics Unit and the Cyprus, School of Molecular Medicine, Cyprus Institute of Neurology & Genetics, Nicosia, Cyprus; 9 Clalit National Cancer Control Center, Carmel Medical Center and Technion Faculty of Medicine, Haifa, Israel; 10 Fred A. Litwin Center for Cancer Genetics, Lunenfeld-Tanenbaum Research Institute of Mount Sinai Hospital, Toronto, ON, Canada; 11 Department of Molecular Genetics, University of Toronto, Toronto, ON, Canada; 12 Department of Epidemiology, Genetic Epidemiology Research Institute, University of California Irvine, Irvine, CA, USA; 13 Division of Clinical Epidemiology and Aging Research, C070, German Cancer Research Center (DKFZ), Heidelberg, Germany; 14 Department of Public Health Sciences and Cancer Research Institute, Queen’s University, Kingston, ON, Canada; 15 Cancer Prevention Program, Fred Hutchinson Cancer Research Center, Seattle, WA, USA; 16 Zilber School of Public Health, University of Wisconsin-Milwaukee, Milwaukee, WI, USA; 17 Institute of Medical Biometry and Epidemiology, University Medical Center Hamburg-Eppendorf, Hamburg, Germany; 18 Institute of Biometry and Clinical Epidemiology, Charité-Universitätsmedizin Berlin, Berlin, Germany; 19 Department of Gynecology and Obstetrics, Comprehensive Cancer Center ER-EMN, University Hospital Erlangen, Friedrich-Alexander-University Erlangen-Nuremberg, Erlangen, Germany; 20 Division of Epidemiology, Department of Medicine, Vanderbilt Epidemiology Center, Vanderbilt-Ingram Cancer Center, Vanderbilt University School of Medicine, Nashville, TN, USA; 21 Centro de Investigación en Red de Enfermedades Raras (CIBERER), Madrid, Spain; 22 Human Cancer Genetics Programme, Spanish National Cancer Research Centre (CNIO), Madrid, Spain; 23 Department of Population Sciences, Beckman Research Institute of City of Hope, Duarte, CA, USA; 24 Copenhagen General Population Study, Herlev and Gentofte Hospital, Copenhagen University Hospital, Herlev, Denmark; 25 Department of Clinical Biochemistry, Herlev and Gentofte Hospital, Copenhagen University Hospital, Herlev, Denmark; 26 Faculty of Health and Medical Sciences, University of Copenhagen, Copenhagen, Denmark; 27 Dr Margarete Fischer-Bosch-Institute of Clinical Pharmacology, Stuttgart, Germany; 28 iFIT-Cluster of Excellence, University of Tuebingen, Tuebingen, Germany; 29 German Cancer Consortium (DKTK), German Cancer Research Center (DKFZ), Heidelberg, Germany; 30 Division of Preventive Oncology, German Cancer Research Center (DKFZ) and National Center for Tumor Diseases (NCT), Heidelberg, Germany; 31 Institute for Prevention and Occupational Medicine of the German Social Accident Insurance, Institute of the Ruhr University Bochum (IPA), Bochum, Germany; 32 Department of Biology, University of Pisa, Pisa, Italy; 33 Genomic Epidemiology Group, German Cancer Research Center (DKFZ), Heidelberg, Germany; 34 Genomic Medicine Group, Galician Foundation of Genomic Medicine, Instituto de Investigación Sanitaria de Santiago de Compostela (IDIS), Complejo Hospitalario Universitario de Santiago, SERGAS, Santiago de Compostela, Spain; 35 Centro de Investigación en Red de Enfermedades Raras (CIBERER) y Centro Nacional de Genotipado (CEGEN-PRB2), Universidad de Santiago de Compostela, Santiago De Compostela, Spain; 36 Behavioral and Epidemiology Research Group, American Cancer Society, Atlanta, GA, USA; 37 Oncology and Genetics Unit, Instituto de Investigacion Sanitaria Galicia Sur (IISGS), Xerencia de Xestion Integrada de Vigo-SERGAS, Vigo, Spain; 38 Department of Biostatistics, Bloomberg School of Public Health, Johns Hopkins University, Baltimore, MD, USA; 39 Department of Oncology, School of Medicine, Johns Hopkins University, Baltimore, MD, USA; 40 Department of Genetics and Computational Biology, QIMR Berghofer Medical Research Institute, Brisbane, Queensland, Australia; 41 Westmead Institute for Medical Research, University of Sydney, Sydney, New South Wales, Australia; 42 Department of Laboratory Medicine and Pathology, Mayo Clinic, Rochester, MN, USA; 43 Sheffield Institute for Nucleic Acids (SInFoNiA), Department of Oncology and Metabolism, University of Sheffield, Sheffield, UK; 44 Academic Unit of Pathology, Department of Neuroscience, University of Sheffield, Sheffield, UK; 45 Department of Medical Epidemiology and Biostatistics, Karolinska Institutet, Stockholm, Sweden; 46 Lineberger Comprehensive Cancer Center, University of North Carolina at Chapel Hill, Chapel Hill, NC, USA; 47 Institute of Human Genetics, University Hospital Erlangen, Friedrich-Alexander University Erlangen-Nuremberg, Comprehensive Cancer Center Erlangen-EMN, Erlangen, Germany; 48 Channing Division of Network Medicine, Department of Medicine, Brigham and Women’s Hospital and Harvard Medical School, Boston, MA, USA; 49 Department of Epidemiology, Harvard T.H. Chan School of Public Health, Boston, MA, USA; 50 Division of Evolution and Genomic Sciences, School of Biological Sciences, Faculty of Biology, Medicine and Health, University of Manchester, Manchester Academic Health Science Centre, Manchester, UK; 51 North West Genomics Laboratory Hub, Manchester Centre for Genomic Medicine, St Mary’s Hospital, Manchester University NHS Foundation Trust, Manchester Academic Health Science Centre, Manchester, UK; 52 David Geffen School of Medicine, Department of Medicine Division of Hematology and Oncology, University of California at Los Angeles, Los Angeles, CA, USA; 53 Usher Institute of Population Health Sciences and Informatics, The University of Edinburgh Medical School, Edinburgh, UK; 54 Cancer Research UK Edinburgh Centre, Edinburgh, UK; 55 School of Public Health, Curtin University, Perth, Western Australia, Australia; 56 Moores Cancer Center, University of California San Diego, La Jolla, CA, USA; 57 Program in Genetic Epidemiology and Statistical Genetics, Harvard T.H. Chan School of Public Health, Boston, MA, USA; 58 Cancer Epidemiology Division, Cancer Council Victoria, Melbourne, Victoria, Australia; 59 Centre for Epidemiology and Biostatistics, Melbourne School of Population and Global Health, The University of Melbourne, Melbourne, Victoria, Australia; 60 Precision Medicine, School of Clinical Sciences at Monash Health, Monash University, Clayton, Victoria, Australia; 61 Cancer & Environment Group, Center for Research in Epidemiology and Population Health (CESP), INSERM, University Paris-Sud, University Paris-Saclay, Villejuif, France; 62 Department of Gynaecology and Obstetrics, University Hospital Erlangen, Friedrich-Alexander University Erlangen-Nuremberg, Comprehensive Cancer Center Erlangen-EMN, Erlangen, Germany; 63 Department of Preventive Medicine, Keck School of Medicine, University of Southern California, Los Angeles, CA, USA; 64 Institute of Environmental Medicine, Karolinska Institutet, Stockholm, Sweden; 65 Department of Oncology, Södersjukhuset, Stockholm, Sweden; 66 Molecular Genetics of Breast Cancer, German Cancer Research Center (DKFZ), Heidelberg, Germany; 67 Laboratory of Experimental Oncology (LEO), Department of Oncology, Leuven Cancer Institute, KU Leuven, Leuven, Belgium; 68 School of Population and Global Health, The University of Western Australia, Perth, Western Australia, Australia; 69 Saarland Cancer Registry, Saarbrücken, Germany; 70 Division of Cancer Sciences, University of Manchester, Manchester, UK; 71 Nuffield Department of Population Health, University of Oxford, Oxford, UK; 72 Australian Breast Cancer Tissue Bank, Westmead Institute for Medical Research, University of Sydney, Sydney, New South Wales, Australia; 73 Research Department, Peter MacCallum Cancer Center, Melbourne, Victoria, Australia; 74 Sir Peter MacCallum Department of Oncology, The University of Melbourne, Melbourne, Victoria, Australia; 75 Division of Oncology, Departments of Epidemiology & Population Health and of Medicine, Stanford Cancer Institute, Stanford University School of Medicine, Stanford, CA, USA; 76 Division of Genetics and Epidemiology, The Institute of Cancer Research, London, UK; 77 Division of Molecular Pathology, The Netherlands Cancer Institute - Antoni van Leeuwenhoek Hospital, Amsterdam, The Netherlands; 78 Radiation Epidemiology Branch, Division of Cancer Epidemiology and Genetics, National Cancer Institute, Bethesda, MD, USA; 79 Department of Internal Medicine, Evangelische Kliniken Bonn gGmbH, Johanniter Krankenhaus, Bonn, Germany; 80 VIB Center for Cancer Biology, VIB, Leuven, Belgium; 81 Laboratory for Translational Genetics, Department of Human Genetics, University of Leuven, Leuven, Belgium; 82 Epidemiology Program, University of Hawaii Cancer Center, Honolulu, HI, USA; 83 Department of Cancer Epidemiology and Prevention, M. Sklodowska-Curie National Research Institute of Oncology, Warsaw, Poland; 84 General and Gastroenterology Surgery Service, Hospital Universitario Central de Asturias, Oviedo, Spain; 85 Department of Family Medicine and Public Health, University of California San Diego, La Jolla, CA, USA; 86 Cancer Epidemiology Group, University Cancer Center Hamburg (UCCH), University Medical Center Hamburg-Eppendorf, Hamburg, Germany; 87 Anatomical Pathology, The Alfred Hospital, Melbourne, Victoria, Australia; 88 Department of Health Sciences Research, Mayo Clinic, Rochester, MN, USA; 89 Epidemiology Branch, National Institute of Environmental Health Sciences, NIH, Research Triangle Park, NC, USA; 90 Department of Epidemiology, Gillings School of Global Public Health and UNC Lineberger Comprehensive Cancer Center, University of North Carolina at Chapel Hill, Chapel Hill, NC, USA; 91 Department of Cancer Epidemiology, Clinical Sciences, Lund University, Lund, Sweden; 92 Centre for Cancer Research and Cell Biology, Queen’s University Belfast, Belfast, Ireland, UK; 93 Department of Genetics, Lineberger Comprehensive Cancer Center, University of North Carolina at Chapel Hill, Chapel Hill, NC, USA; 94 Human Genotyping-CEGEN Unit, Human Cancer Genetic Program, Spanish National Cancer Research Centre, Madrid, Spain; 95 Department of Oncology, Mayo Clinic, Rochester, MN, USA; 96 School of Medicine, University of Western Australia, Perth, Western Australia, Australia; 97 Network Aging Research, University of Heidelberg, Heidelberg, Germany; 98 Department of Population and Quantitative Health Sciences, Case Western Reserve University, Cleveland, OH, USA; 99 Division of Molecular Medicine, Pathology North, John Hunter Hospital, Newcastle, New South Wales, Australia; 100 Discipline of Medical Genetics, School of Biomedical Sciences and Pharmacy, Faculty of Health, University of Newcastle, Callaghan, New South Wales, Australia; 101 Hunter Medical Research Institute, John Hunter Hospital, Newcastle, New South Wales, Australia; 102 Department of Surgical Oncology, University Hospitals Leuven, Leuven, Belgium; 103 Department of Clinical Pathology, The University of Melbourne, Melbourne, Victoria, Australia; 104 Cancer Epidemiology Centre, Cancer Council Victoria, Melbourne, Victoria, Australia; 105 Population Oncology, BC Cancer, Vancouver, BC, Canada; 106 School of Population and Public Health, University of British Columbia, Vancouver, BC, Canada; 107 The Curtin UWA Centre for Genetic Origins of Health and Disease, Curtin University and University of Western Australia, Perth, Western Australia, Australia; 108 Division of Breast Cancer Research, The Institute of Cancer Research, London, UK; 109 Epigenetic and Stem Cell Biology Laboratory, National Institute of Environmental Health Sciences, NIH, Research Triangle Park, NC, USA; 110 Department of Health Science Research, Division of Epidemiology, Mayo Clinic, Rochester, MN, USA; 111 Biostatistics and Computational Biology Branch, National Institute of Environmental Health Sciences, NIH, Research Triangle Park, NC, USA; 112 Leuven Multidisciplinary Breast Center, Department of Oncology, Leuven Cancer Institute, University Hospitals Leuven, Leuven, Belgium; 113 Department of Nutrition, Harvard T.H. Chan School of Public Health, Boston, MA, USA; 114 Channing Division of Network Medicine, Brigham and Women’s Hospital and Harvard Medical School, Boston, MA, USA; 115 Division of Biomedical Statistics and Informatics, Department of Health Science Research, Mayo Clinic, Rochester, MN, USA; 116 Department of Surgical Sciences, Uppsala University, Uppsala, Sweden; 117 Centre for Cancer Genetic Epidemiology, Department of Oncology, University of Cambridge, Cambridge, UK; 118 Division of Psychosocial Research and Epidemiology, The Netherlands Cancer Institute - Antoni van Leeuwenhoek Hospital, Amsterdam, The Netherlands

## Abstract

We evaluated the joint associations between a new 313-variant PRS (PRS_313_) and questionnaire-based breast cancer risk factors for women of European ancestry, using 72 284 cases and 80 354 controls from the Breast Cancer Association Consortium. Interactions were evaluated using standard logistic regression and a newly developed case-only method for breast cancer risk overall and by estrogen receptor status. After accounting for multiple testing, we did not find evidence that per-standard deviation PRS_313_ odds ratio differed across strata defined by individual risk factors. Goodness-of-fit tests did not reject the assumption of a multiplicative model between PRS_313_ and each risk factor. Variation in projected absolute lifetime risk of breast cancer associated with classical risk factors was greater for women with higher genetic risk (PRS_313_ and family history) and, on average, 17.5% higher in the highest vs lowest deciles of genetic risk. These findings have implications for risk prevention for women at increased risk of breast cancer.

Precision prevention and early detection of cancer is a key aim of cancer research and uses tools such as risk prediction models for risk stratification (1,2). Many breast cancer risk prediction models are focused either on classical risk factors or on inherited mutations causing a moderate-to-high risk of cancer and do not include risk associated with common susceptibility variants (3). Modeling the joint associations of genetic and classical risk factors could result in substantial improvement in risk stratification and therefore improved prevention and screening modalities for breast cancer (4–7).

Combined associations of single nucleotide polymorphisms (SNPs) can be summarized by a polygenic risk score (PRS); women in the top 1% of the newly derived 313-SNP PRS (PRS_313_) have a fourfold increased risk of breast cancer than women at population-average risk (8). Previous studies, which evaluated combined associations between classical risk factors and breast cancer PRS based on 77 SNPs (9) and 24 SNPs (10), found weak or no evidence of departure from the multiplicative risk assumption for overall breast cancer. In the current study, we extend these analyses to assess the combined associations of the PRS_313_ and classical risk factors using data from the Breast Cancer Association Consortium (BCAC). This new PRS has been validated by prospective studies and shown to be more predictive than the previously reported 77-SNP PRS (11) for risk of breast cancer overall as well as for estrogen receptor (ER) subtype-specific breast cancer (8). Additionally, this study found evidence of interaction for ER-positive disease between PRS_313_ and family history, indicating the need to consider the joint effects of these 2 factors (8).

Detailed information on study samples, genetic data, and risk factor data is provided in the Supplementary Materials (available online). Briefly, we performed analyses using data from women of European ancestry from 16 prospective cohorts, 14 population-based case-control studies, and 16 nonpopulation-based studies included in BCAC (Supplementary Table 1, available online). Samples were genotyped using 2 arrays: iCOGS (12) and OncoArray (13–15). Risk factor data were derived with respect to a reference age (date at diagnosis for cases and date at interview for controls). Development of the PRS is briefly explained in Supplementary Materials (available online) (8). We standardized the PRS to have unit standard deviation for the controls.

Departure from the assumption of multiplicative combined effects of standardized PRS_313_ and each risk factor was assessed using two methods: unconditional logistic regression model and likelihood ratio test, and a newly developed case-only method, which assumes independence between PRS and risk factors in the underlying population and has greater efficiency compared with logistic regression (16). Individual models were fitted for each PRS-risk factor combination for overall and ER-specific breast cancer. Models were adjusted for reference age, study, and corresponding 10 ancestry-informative principal components for each array. Array-specific results were meta-analyzed using a fixed-effect inverse-variance weighted method. To evaluate global goodness-of-fit of the multiplicative model between PRS_313_ and each risk factor, we performed the Hosmer-Lemeshow test using population-based studies. Moreover, we assessed goodness-of-fit at the extremes of the distribution (tails) using a tail-based test (17). Using the iCARE-BPC3 model (4), we projected absolute lifetime risk of breast cancer for 50-year-old white non-Hispanic US women up to aged 80 years. We assessed the distribution of risk because of classical (ie, menstrual and reproductive and lifestyle) and modifiable risk factors, respectively, within categories of risk defined by genetic factors (ie, breast cancer family history and PRS_313_).

Associations between PRS_313_ and overall and ER-specific breast cancer risk are likely to be overestimated because there was substantial overlap between the SNP discovery samples and our dataset (Supplementary Figure 1, available online). The number of cases and controls varied for each risk factor, ranging from 61 617 cases and 74 698 controls for ever parous to 14 576 cases and 19 640 controls for pack-years smoked for overall breast cancer risk (Supplementary Table 2, available online). Based on the population-based case-control and prospective cohort studies, the associations of the risk factors with overall and ER subtype-specific breast cancer were of the expected magnitude and direction (Supplementary Table 3, available online).

After accounting for multiple testing using Bonferroni adjustment (*P*_int_ < .05/16 = .003), none of the interactions between PRS_313_ and any classical risk factor was statistically significant except for family history (Table 1). All statistical tests were 2-sided. The observed interaction between PRS_313_ and family history for ER-positive breast disease is consistent with what has been previously published based on an overlapping dataset (8). Such an interaction was also found for overall and ER-negative breast cancer risk. There was no evidence for a clear dose-response in the estimated ORs associated with classical risk factors when stratified by PRS percentiles (Supplementary Figures 2–4, available online). Neither global nor tail-based goodness-of-fit tests supported departure from the multiplicative model for any risk factor for both overall and ER-positive breast cancer (Supplementary Table 4, available online). Goodness-of-fit tests were not performed for ER-negative breast cancer because of the relatively small sample size.

**Table 1.** Odds ratios and 95% confidence intervals for multiplicative interactions between the 313-SNP PRS (continuous) and classical risk factors of breast cancer, overall and by ER status

Risk factors	Controls	Case-control logistic regression method^a^^,c^									Case-only linear regression method^a^^,^^b^^,^^c^					
		Overall breast cancer risk			ER-positive breast cancer risk			ER-negative breast cancer risk			Overall breast cancer risk		ER-positive breast cancer risk		ER-negative breast cancer risk	
		Cases	OR_int_ (95% CI)	*P*_int_	Cases	OR_int_ (95% CI)	*P*_int_	Cases	OR_int_ (95% CI)	*P*_int_	OR_int_ (95% CI)	*P*_int_	OR_int_ (95% CI)	*P*_int_	OR_int_ (95% CI)	*P*_int_
Age at menarche (per 2 years)	64 087	52 170	1.01 (0.99 to 1.03)	.26	36 820	1.01 (0.99 to 1.03)	.29	8323	1.01 (0.98 to 1.04)	.55	1.01 (1.00 to 1.02)	.22	1.01 (0.99 to 1.02)	.37	1.02 (0.99 to 1.06)	.21
Ever parous (yes/no)	72 552	59 298	0.97 (0.93 to 1.00)	.07	41 858	0.98 (0.94 to 1.02)	.35	9273	0.98 (0.92 to 1.05)	.57	0.97 (0.94 to 1.00)	.08	0.99 (0.96 to 1.03)	.72	1.00 (0.92 to 1.09)	.97
Number of children (1, 2, 3, ≥4)^d^	61 654	48 786	1.00 (0.99 to 1.02)	.96	34 666	1.00 (0.99 to 1.02)	.73	7552	0.99 (0.96 to 1.02)	.53	1.01 (0.99 to 1.02)	.38	1.01 (1.00 to 1.03)	.11	1.00 (0.97 to 1.04)	.90
Age at FFTP (per 5 years)^d^	53 042	41 671	1.00 (0.99 to 1.02)	.82	29 601	1.00 (0.98 to 1.01)	.68	6517	0.99 (0.96 to 1.02)	.52	1.00 (0.98 to 1.01)	.39	0.99 (0.97 to 1.00)	.06	1.00 (0.97 to 1.03)	.92
Breastfeeding (yes/no)^d^	37 568	34 199	1.02 (0.98 to 1.06)	.44	24 273	1.01 (0.96 to 1.05)	.81	5548	1.01 (0.95 to 1.08)	.74	1.02 (0.99 to 1.05)	.17	1.02 (0.98 to 1.06)	.36	1.02 (0.95 to 1.11)	.55
Duration of breast feeding (per 12 mo)^d^	26 367	27 741	1.00 (0.98 to 1.02)	.71	19 329	1.00 (0.97 to 1.02)	.76	4669	0.99 (0.95 to 1.03)	.57	1.01 (0.99 to 1.03)	.32	1.01 (0.99 to 1.03)	.57	0.99 (0.96 to 1.03)	.77
Adult height (per 5 cm)	62 414	54 847	0.99 (0.98 to 1.00)	.07	38 730	0.99 (0.98 to 1.00)	.04	8682	1.00 (0.98 to 1.02)	.77	1.00 (0.99 to 1.01)	.92	0.99 (0.98 to 1.01)	.29	1.01 (0.99 to 1.03)	.48
Premenopausal BMI (per 5 kg/m^2^)^e^	15 610	12 837	0.98 (0.95 to 1.00)	.08	8354	0.99 (0.96 to 1.02)	.48	2333	0.95 (0.91 to 1.00)	.04	0.97 (0.94 to 1.00)	.02	1.00 (0.96 to 1.03)	.77	0.95 (0.89 to 1.01)	.10
Postmenopausal BMI (per 5 kg/m^2^)^f^	46 137	37 088	1.01 (0.99 to 1.02)	.49	27 305	1.01 (0.99 to 1.02)	.39	5260	1.01 (0.99 to 1.04)	.36	1.01 (1.00 to 1.02)	.29	1.01 (1.00 to 1.03)	.08	0.99 (0.96 to 1.02)	.45
Ever use of oral contraceptives (yes/no)	56 768	44 979	1.01 (0.98 to 1.04)	.63	31 640	1.02 (0.98 to 1.05)	.36	7061	1.02 (0.97 to 1.08)	.42	0.99 (0.97 to 1.02)	.45	1.00 (0.97 to 1.02)	.75	1.01 (0.95 to 1.08)	.73
Current use of EPT (yes/no)^f^^,^^g^	20 896	19 047	1.07 (1.01 to 1.14)	.02	14 465	1.06 (0.99 to 1.13)	.08	2761	1.05 (0.92 to 1.19)	.49	1.00 (0.96 to 1.04)	.93	0.98 (0.93 to 1.03)	.32	1.04 (0.91 to 1.18)	.59
Current use of ET (yes/no)^f^^,^^g^	20 716	18 716	0.97 (0.91 to 1.03)	.33	14 201	0.96 (0.90 to 1.03)	.28	2733	1.06 (0.94 to 1.20)	.37	0.96 (0.91 to 1.01)	.09	0.94 (0.89 to 0.99)	.03	1.08 (0.95 to 1.23)	.26
Alcohol consumption (per 10 g/day)	16 851	14 484	1.00 (0.97 to 1.02)	.75	10 253	0.98 (0.96 to 1.00)	.07	2259	1.06 (1.01 to 1.11)	.03	1.00 (0.99 to 1.02)	.71	0.99 (0.97 to 1.01)	.19	1.04 (1.00 to 1.08)	.06
Current smoking (yes/no)^h^	56 308	43 303	1.04 (1.00 to 1.08)	.07	30 486	1.05 (1.00 to 1.10)	.03	6813	1.05 (0.97 to 1.13)	.25	1.02 (0.98 to 1.05)	.42	1.02 (0.98 to 1.06)	.40	1.03 (0.95 to 1.11)	.52
Pack-years of smoking (per 10 pack-years)^i^	15 990	11 766	0.99 (0.98 to 1.01)	.43	8268	0.99 (0.97 to 1.01)	.19	1778	0.99 (0.96 to 1.02)	.67	1.00 (0.99 to 1.01)	.97	1.00 (0.99 to 1.01)	.99	1.00 (0.97 to 1.03)	.97
Family history in a first-degree relative (yes/no)^j^	50 955	42 024	0.93 (0.89 to 0.96)	3.00 × 10^−5^	28 909	0.93 (0.90 to 0.97)	8.00 × 10^−4^	6921	0.93 (0.87 to 0.99)	.03	—	—	—	—	—	—

^a^ Number of cases are same for case-control and case-only method. PRS = polygenic risk score; SNP = single nucleotide polymorphisms; ORint = interaction odds ratio (per SD of PRS_313_); CI = confidence intervals; FFTP = first full-term pregnancy; BMI = body mass index; EPT = estrogen-progesterone therapy; ET = estrogen-only therapy; ER = estrogen receptor; MHT = menopausal hormonal therapy.

^b^ The case-only analyses do not provide additional evidence to case-control analyses.

^c^ Models are adjusted for reference age, study, and 10 ancestry-informative principal components.

^d^ Among parous women.

^e^ Among premenopausal women.

^f^ Among postmenopausal women.

^g^ Models used to assess association with the use of MHT have been further adjusted for former use of MHT and use of any other type MHT preparations.

^h^ Models used to assess association with current smoking have been further adjusted for former smoking.

^i^ Among ever smoked.

^j^ PRS and family history are not independent; therefore, case-only analyses were not conducted for family history.

Lack of evidence for substantial departure from the multiplicative assumption between the PRS_313_ and risk factors using this large study implies that the absolute risk associated with each classical risk factor is greater for women with higher polygenic risk (5,18). This is illustrated by our projections, which show that the lifetime risk due to classical risk factors was higher with a wider variation across women who are at a higher risk due to genetic factors (PRS_313_ and family history) (Figure 1, a) and consistent with a recent study of body mass index combined with a measure of familial risk based on multigenerational family history (18). The predicted average lifetime risk due to all classical risk factors for women in the lowest and highest deciles of the genetic risk was 21.9% and 4.4%, respectively, so the difference in risk was 17.5%. The difference in risk between these 2 deciles associated with the subset of modifiable risk factors was 16.5% (Figure 1, b). However, the absolute risk projections shown in Figure 1 should be viewed with caution because they assume perfect model calibration. In addition, these absolute risk projections require validation.

**Figure 1.**
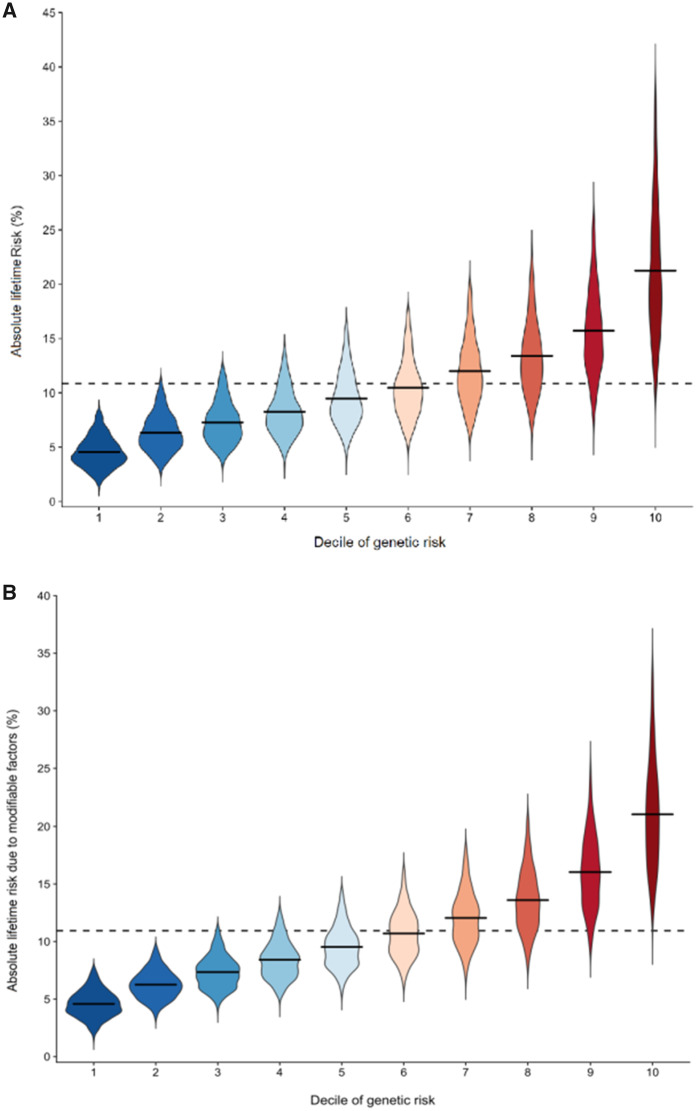
Distribution of absolute lifetime risk explained by **(a)** all classical risk factors and **(b)** modifiable classical risk factors within decile categories of genetic risk, due to 313-variant polygenic risk score (PRS) and family history, for 50-year-old white non-Hispanic women in the United States before aged 80 years. The **solid horizontal lines** represent the mean risk within each decile, and the **dashed horizontal line** across the plot represents the population lifetime mean risk (10.9%). Lifetime risk is estimated using the iCARE-BPC3 model and refers to absolute risk from aged 50 to 80 years. The genetic component includes the 313-variant PRS and breast cancer family history. The classical risk factor component includes the following risk factors: age at menarche, age at menopause, parity, age at first birth, height, body mass index (BMI), alcohol intake, smoking status, ever and current use of hormone replacement therapy (HRT), and HRT type among ever users. The modifiable classical risk factor component includes BMI, ever or current use of HRT, smoking status, and alcohol consumption. Outliers defined as points beyond 1.5 times the interquartile range below the first quartile or above the third quartile were excluded from the plot.

Our analyses using the current PRS_313_ are based on a sample size 3 times larger than that used in previously published BCAC analyses (9), although the dataset for ER-negative breast cancer is still limited. Our previous work on the PRS_313_ development (8) and the current analyses is based on European ancestry and may not be generalizable to other populations, highlighting the need for more studies in populations of non-European or mixed ancestry.

Overall, the combined associations of the newly developed PRS_313_ and the classical risk factors on breast cancer risk are well explained by a multiplicative model, except for family history, and will inform the development of overall and ER-specific risk prediction models in the future. Most important, our findings suggest that preventive strategies aimed at modifying individual risk factors could have stronger impact on absolute risk reduction for women at higher genetic risk.

## Funding

This work was supported by following funding agencies. BCAC is funded by Cancer Research UK [C1287/A16563, C1287/A10118], the European Union’s Horizon 2020 Research and Innovation Programme (grant numbers 634935 and 633784 for BRIDGES and B-CAST respectively), and by the European Communitýs Seventh Framework Programme under grant agreement number 223175 (grant number HEALTH-F2-2009–223175) (COGS). The EU Horizon 2020 Research and Innovation Programme funding source had no role in study design, data collection, data analysis, data interpretation or writing of the report.

Genotyping of the OncoArray was funded by the NIH Grant U19 CA148065, and Cancer UK Grant C1287/A16563 and the PERSPECTIVE project supported by the Government of Canada through Genome Canada and the Canadian Institutes of Health Research (grant GPH-129344) and, the Ministère de l’Économie, Science et Innovation du Québec through Genome Québec and the PSRSIIRI-701 grant, and the Quebec Breast Cancer Foundation. Funding for the iCOGS infrastructure came from: the European Community’s Seventh Framework Programme under grant agreement n° 223175 (HEALTH-F2-2009–223175) (COGS), Cancer Research UK (C1287/A10118, C1287/A10710, C12292/A11174, C1281/A12014, C5047/A8384, C5047/A15007, C5047/A10692, C8197/A16565), the National Institutes of Health (CA128978) and Post-Cancer GWAS initiative (1U19 CA148537, 1U19 CA148065 and 1U19 CA148112 - the GAME-ON initiative), the Department of Defence (W81XWH-10–1-0341), the Canadian Institutes of Health Research (CIHR) for the CIHR Team in Familial Risks of Breast Cancer, and Komen Foundation for the Cure, the Breast Cancer Research Foundation, and the Ovarian Cancer Research Fund. The DRIVE Consortium was funded by U19 CA148065.

The Australian Breast Cancer Family Study (ABCFS) was supported by grant UM1 CA164920 from the National Cancer Institute (USA). The content of this manuscript does not necessarily reflect the views or policies of the National Cancer Institute or any of the collaborating centers in the Breast Cancer Family Registry (BCFR), nor does mention of trade names, commercial products, or organizations imply endorsement by the USA Government or the BCFR. The ABCFS was also supported by the National Health and Medical Research Council of Australia, the New South Wales Cancer Council, the Victorian Health Promotion Foundation (Australia) and the Victorian Breast Cancer Research Consortium. J.L.H. is a National Health and Medical Research Council (NHMRC) Senior Principal Research Fellow. M.C.S. is a NHMRC Senior Research Fellow. The ABCS study was supported by the Dutch Cancer Society [grants NKI 2007–3839; 2009 4363]. The Australian Breast Cancer Tissue Bank (ABCTB) was supported by the National Health and Medical Research Council of Australia, The Cancer Institute NSW and the National Breast Cancer Foundation. The AHS study is supported by the intramural research program of the National Institutes of Health, the National Cancer Institute (grant number Z01-CP010119), and the National Institute of Environmental Health Sciences (grant number Z01-ES049030). The work of the BBCC was partly funded by ELAN-Fond of the University Hospital of Erlangen. The BCEES was funded by the National Health and Medical Research Council, Australia and the Cancer Council Western Australia and acknowledges funding from the National Breast Cancer Foundation (JS). The BREast Oncology GAlician Network (BREOGAN) is funded by Acción Estratégica de Salud del Instituto de Salud Carlos III FIS PI12/02125/Cofinanciado FEDER; Acción Estratégica de Salud del Instituto de Salud Carlos III FIS Intrasalud (PI13/01136); Programa Grupos Emergentes, Cancer Genetics Unit, Instituto de Investigacion Biomedica Galicia Sur. Xerencia de Xestion Integrada de Vigo-SERGAS, Instituto de Salud Carlos III, Spain; Grant 10CSA012E, Consellería de Industria Programa Sectorial de Investigación Aplicada, PEME I + D e I + D Suma del Plan Gallego de Investigación, Desarrollo e Innovación Tecnológica de la Consellería de Industria de la Xunta de Galicia, Spain; Grant EC11-192. Fomento de la Investigación Clínica Independiente, Ministerio de Sanidad, Servicios Sociales e Igualdad, Spain; and Grant FEDER-Innterconecta. Ministerio de Economia y Competitividad, Xunta de Galicia, Spain. CBCS is funded by the Canadian Cancer Society (grant # 313404) and the Canadian Institutes of Health Research. The CECILE study was supported by Fondation de France, Institut National du Cancer (INCa), Ligue Nationale contre le Cancer, Agence Nationale de Sécurité Sanitaire, de l’Alimentation, de l’Environnement et du Travail (ANSES), Agence Nationale de la Recherche (ANR). The CGPS was supported by the Chief Physician Johan Boserup and Lise Boserup Fund, the Danish Medical Research Council, and Herlev and Gentofte Hospital. The CNIO-BCS was supported by the Instituto de Salud Carlos III, the Red Temática de Investigación Cooperativa en Cáncer and grants from the Asociación Española Contra el Cáncer and the Fondo de Investigación Sanitario (PI11/00923 and PI12/00070). The American Cancer Society funds the creation, maintenance, and updating of the CPS-II cohort. The CTS was supported by the California Breast Cancer Act of 1993, the California Breast Cancer Research Fund (contract 97–10500) and the National Institutes of Health (R01 CA77398, K05 CA136967, UM1 CA164917, and U01 CA199277). Collection of cancer incidence data was supported by the California Department of Public Health as part of the statewide cancer reporting program mandated by California Health and Safety Code Section 103885. HAC receives support from the Lon V Smith Foundation (LVS39420). The coordination of EPIC is financially supported by the European Commission (DG-SANCO) and the International Agency for Research on Cancer. The national cohorts are supported by: Ligue Contre le Cancer, Institut Gustave Roussy, Mutuelle Générale de l’Education Nationale, Institut National de la Santé et de la Recherche Médicale (INSERM) (France); German Cancer Aid, German Cancer Research Center (DKFZ), Federal Ministry of Education and Research (BMBF) (Germany); the Hellenic Health Foundation, the Stavros Niarchos Foundation (Greece); Associazione Italiana per la Ricerca sul Cancro-AIRC-Italy and National Research Council (Italy); Dutch Ministry of Public Health, Welfare and Sports (VWS), Netherlands Cancer Registry (NKR), LK Research Funds, Dutch Prevention Funds, Dutch ZON (Zorg Onderzoek Nederland), World Cancer Research Fund (WCRF), Statistics Netherlands (The Netherlands); Health Research Fund (FIS), PI13/00061 to Granada, PI13/01162 to EPIC-Murcia, Regional Governments of Andalucía, Asturias, Basque Country, Murcia and Navarra, ISCIII RETIC (RD06/0020) (Spain); Cancer Research UK (14136 to EPIC-Norfolk; C570/A16491 and C8221/A19170 to EPIC-Oxford), Medical Research Council (1000143 to EPIC-Norfolk, MR/M012190/1 to EPIC-Oxford) (United Kingdom). The ESTHER study was supported by a grant from the Baden Württemberg Ministry of Science, Research and Arts. Additional cases were recruited in the context of the VERDI study, which was supported by a grant from the German Cancer Aid (Deutsche Krebshilfe). The GENICA was funded by the Federal Ministry of Education and Research (BMBF) Germany grants 01KW9975/5, 01KW9976/8, 01KW9977/0 and 01KW0114, the Robert Bosch Foundation, Stuttgart, Deutsches Krebsforschungszentrum (DKFZ), Heidelberg, the Institute for Prevention and Occupational Medicine of the German Social Accident Insurance, Institute of the Ruhr University Bochum (IPA), Bochum, as well as the Department of Internal Medicine, Evangelische Kliniken Bonn gGmbH, Johanniter Krankenhaus, Bonn, Germany. The GESBC was supported by the Deutsche Krebshilfe e. V. [70492] and the German Cancer Research Center (DKFZ). The KARMA study was supported by Märit and Hans Rausings Initiative Against Breast Cancer. kConFab is supported by a grant from the National Breast Cancer Foundation, and previously by the National Health and Medical Research Council (NHMRC), the Queensland Cancer Fund, the Cancer Councils of New South Wales, Victoria, Tasmania and South Australia, and the Cancer Foundation of Western Australia. Financial support for the AOCS was provided by the United States Army Medical Research and Materiel Command [DAMD17-01–1-0729], Cancer Council Victoria, Queensland Cancer Fund, Cancer Council New South Wales, Cancer Council South Australia, The Cancer Foundation of Western Australia, Cancer Council Tasmania and the National Health and Medical Research Council of Australia (NHMRC; 400413, 400281, 199600). G.C.T. and P.W. are supported by the NHMRC. RB was a Cancer Institute NSW Clinical Research Fellow. LMBC is supported by the ‘Stichting tegen Kanker’. DL is supported by the FWO. The MARIE study was supported by the Deutsche Krebshilfe e. V. [70–2892-BR I, 106332, 108253, 108419, 110826, 110828], the Hamburg Cancer Society, the German Cancer Research Center (DKFZ) and the Federal Ministry of Education and Research (BMBF) Germany [01KH0402]. The MCBCS was supported by the NIH grants CA192393, CA116167, CA176785 an NIH Specialized Program of Research Excellence (SPORE) in Breast Cancer [CA116201], and the Breast Cancer Research Foundation and a generous gift from the David F. and Margaret T. Grohne Family Foundation. MCCS cohort recruitment was funded by VicHealth and Cancer Council Victoria. The MCCS was further augmented by Australian National Health and Medical Research Council grants 209057, 396414 and 1074383 and by infrastructure provided by Cancer Council Victoria. Cases and their vital status were ascertained through the Victorian Cancer Registry and the Australian Institute of Health and Welfare, including the National Death Index and the Australian Cancer Database. The MEC was support by NIH grants CA63464, CA54281, CA098758, CA132839 and CA164973. The MISS study is supported by funding from ERC-2011–294576 Advanced grant, Swedish Cancer Society, Swedish Research Council, Local hospital funds, Berta Kamprad Foundation, Gunnar Nilsson. The MMHS study was supported by NIH grants CA97396, CA128931, CA116201, CA140286, and CA177150. The NBHS was supported by NIH grant R01CA100374. Biological sample preparation was conducted the Survey and Biospecimen Shared Resource, which is supported by P30 CA68485. The Northern California Breast Cancer Family Registry (NC-BCFR) and Ontario Familial Breast Cancer Registry (OFBCR) were supported by grant UM1 CA164920 from the National Cancer Institute (USA). The content of this manuscript does not necessarily reflect the views or policies of the National Cancer Institute or any of the collaborating centers in the Breast Cancer Family Registry (BCFR), nor does mention of trade names, commercial products, or organizations imply endorsement by the USA Government or the BCFR. The Carolina Breast Cancer Study was funded by Komen Foundation, the National Cancer Institute (P50 CA058223, U54 CA156733, U01 CA179715), and the North Carolina University Cancer Research Fund. The NHS was supported by NIH grants P01 CA87969, UM1 CA186107, and U19 CA148065. The NHS2 was supported by NIH grants UM1 CA176726 and U19 CA148065 and the Breast Cancer Research Foundation. The PBCS was funded by Intramural Research Funds of the National Cancer Institute, Department of Health and Human Services, USA. Genotyping for PLCO was supported by the Intramural Research Program of the National Institutes of Health, NCI, Division of Cancer Epidemiology and Genetics. The PLCO is supported by the Intramural Research Program of the Division of Cancer Epidemiology and Genetics and supported by contracts from the Division of Cancer Prevention, National Cancer Institute, National Institutes of Health. PROCAS is funded from NIHR grant PGfAR 0707–10031. Prof. Dr D Gareth Evans is supported by the NIHR Biomedical Research Centre in Manchester (IS-BRC-1215–20007). The SASBAC study was supported by funding from the Agency for Science, Technology and Research of Singapore (A*STAR), the US National Institute of Health (NIH) and the Susan G. Komen Breast Cancer Foundation. The SBCS was supported by Sheffield Experimental Cancer Medicine Centre and Breast Cancer Now Tissue Bank. SEARCH is funded by Cancer Research UK [C490/A10124, C490/A16561] and supported by the UK National Institute for Health Research Biomedical Research Centre at the University of Cambridge. The University of Cambridge has received salary support for PDPP from the NHS in the East of England through the Clinical Academic Reserve. The Sister Study (SISTER) is supported by the Intramural Research Program of the NIH, National Institute of Environmental Health Sciences (Z01-ES044005 and Z01-ES049033). The SMC is funded by the Swedish Cancer Foundation and the Swedish Research Council (VR 2017–00644) grant for the Swedish Infrastructure for Medical Population-based Life-course Environmental Research (SIMPLER). The UKBGS is funded by Breast Cancer Now and the Institute of Cancer Research (ICR), London. ICR acknowledges NHS funding to the NIHR Biomedical Research Centre. The USRT Study was funded by Intramural Research Funds of the National Cancer Institute, Department of Health and Human Services, USA. The WHI program is funded by the National Heart, Lung, and Blood Institute, the US National Institutes of Health and the US Department of Health and Human Services (HHSN268201100046C, HHSN268201100001C, HHSN268201100002C, HHSN268201100003C, HHSN268201100004C, and HHSN271201100004C). This work was also funded by NCI U19 CA148065-01.

## Notes

**Role of the funder:** The funders had no role in the design of the study; the collection, analysis, and interpretation of the data; the writing of the manuscript; and the decision to submit the manuscript.

**Disclosures:** The authors declare no conflicts of interest.

**Acknowledgments:** The authors thank all the researchers, clinicians, technicians, and administrative staff involved in the Breast Cancer Association Consortium. The authors would also like to thank all study participants, researchers, clinicians, technicians, and administrative staff who participated in the parent studies (ABCFS, ABCS, ABCTB, AHS, BBCC, BCEES, BCINIS, BREOGAN, CBCS, CECILE, CGPS, CNIO-BCS, CPSII, CTS, EPIC, ESTHER, GENICA, GESBC, KARMA, kConFab /AOCS, LMBC, MARIE, MCBCS, MCCS, MEC, MISS, MMHS, NBHS, NC-BCFR, NCBCS, NHS, NHS2, OFBCR, PBCS, PKARMA, PLCO, PROCAS, SASBAC, SBCS, SEARCH, SISTER, SMC, UCIBCS, UKBGS, USRT, WHI) and have enabled this work to be carried out. The authors thank the staff of the Center for Genetic Epidemiology Laboratory, the CNIO genotyping unit, the McGill University and Génome Québec Innovation Centre, and the Mayo Clinic Genotyping Core Facility. CPSII acknowledges the contribution to this study from central cancer registries supported through the Centers for Disease Control and Prevention National Program of Cancer Registries, as well as cancer registries supported by the National Cancer Institute Surveillance Epidemiology and End Results program. The GENICA Network thanks Dr Margarete Fischer-Bosch-Institute of Clinical Pharmacology, Stuttgart, and University of Tübingen, Germany, German Cancer Consortium (DKTK) and German Cancer Research Center (DKFZ), gefördert durch die Deutsche Forschungsgemeinschaft (DFG) im Rahmen der Exzellenzstrategie des Bundes und der Länder - EXC 2180–390900677, Department of Internal Medicine, Evangelische Kliniken Bonn gGmbH, Johanniter Krankenhaus, Bonn, Germany, Institute of Pathology, University of Bonn, Germany, Molecular Genetics of Breast Cancer, Institute for Prevention and Occupational Medicine of the German Social Accident Insurance, Institute of the Ruhr University Bochum (IPA), Bochum, Germany, and Institute of Occupational Medicine and Maritime Medicine, University Medical Center Hamburg-Eppendorf, Germany. KARMA and SASBAC studies thank the Swedish Medical Research Counsel. kConFab/AOCS wishes to thank all the kConFab research nurses and staff, the heads and staff of the Family Cancer Clinics, and the Clinical Follow Up Study (which has received funding from the NHMRC, the National Breast Cancer Foundation, Cancer Australia, and the National Institute of Health (USA) for their contributions to this resource, and the many families who contribute to kConFab. NHS and NHS2 would like to thank the following state cancer registries for their help: AL, AZ, AR, CA, CO, CT, DE, FL, GA, ID, IL, IN, IA, KY, LA, ME, MD, MA, MI, NE, NH, NJ, NY, NC, ND, OH, OK, OR, PA, RI, SC, TN, TX, VA, WA, WY. PROCAS thanks NIHR for funding. UKBGS thanks Breast Cancer Now and the Institute of Cancer Research for support and funding of the Breakthrough Generations Study. We acknowledge NHS funding to the Royal Marsden/ICR NIHR Biomedical Research Centre.

**Author contributions:** **PMK:** Conceptualization; Data curation; Formal analysis; Investigation; Methodology; Project administration; Validation; Visualization; Writing—original draft; Writing—review & editing. **NM:** Data curation; Methodology; Validation; Writing—review & editing. **PPC:** Methodology; Validation; Visualization; Writing—review & editing. **ANW:** Methodology; Validation; Visualization; Writing—review & editing. **SL:** Data curation; Writing—review & editing. **SB:** Data curation; Writing—review & editing. **KM:** Data curation; Writing—review & editing. **JD:** Data curation; Writing—review & editing. **MKB:** Data curation; Writing—review & editing. **QW:** Data curation; Writing—review & editing. **AJ:** Writing—review & editing. **ZA-F:** Writing—review & editing. **TA:** Writing—review & editing. **ILA:** Data curation; Writing—review & editing. **HAC:** Writing—review & editing. **VA:** Writing—review & editing. **KJA:** Writing—review & editing. **PLA:** Writing—review & editing. **LEBF:** Writing—review & editing. **HB:** Data curation; Writing—review & editing. **MWB:** Writing—review & editing. **AB-F:** Writing—review & editing. **JB:** Writing—review & editing. **LB:** Writing—review & editing. **SEB:** Writing—review & editing. **HB:** Writing—review & editing. **TB:** Writing—review & editing. **QC:** Writing—review & editing. **DC:** Writing—review & editing. **FC:** Writing—review & editing. **AC:** Writing—review & editing. **BDC:** Writing—review & editing. **JEC:** Writing—review & editing. **SJC:** Writing—review & editing. **NC:** Methodology; Writing—review & editing. **GC-T:** Writing—review & editing. **CLC:** Writing—review & editing. **FJC:** Data curation; Writing—review & editing. **AC:** Writing—review & editing. **SSC:** Writing—review & editing. **KC:** Writing—review & editing. **JYD:** Writing—review & editing. **HSE:** Writing—review & editing. **ABE:** Writing—review & editing. **AHE:** Writing—review & editing. **ME:** Writing—review & editing. **DGE:** Writing—review & editing. **PAF:** Writing—review & editing. **JF:** Writing—review & editing. **LF:** Writing—review & editing. **MG:** Writing—review & editing. **MG-D:** Writing—review & editing. **CG:** Writing—review & editing. **SMG:** Writing—review & editing. **MMG:** Writing—review & editing. **GGG:** Writing—review & editing. **AG-N:** Writing—review & editing. **PG:** Writing—review & editing. **LH:** Writing—review & editing. **CAH:** Writing—review & editing. **NH:** Writing—review & editing. **PH:** Data curation; Writing—review & editing. **UH:** Writing—review & editing. **SH:** Writing—review & editing. **JH:** Writing—review & editing. **BH:** Writing—review & editing. **RNH:** Writing—review & editing. **JLH:** Writing—review & editing. **AH:** Writing—review & editing. **DJH:** Writing—review & editing. **EMJ:** Writing—review & editing. **MEJ:** Writing—review & editing. **RK:** Writing—review & editing. **RK:** Writing—review & editing. **CMK:** Writing—review & editing. **Y-DK:** Writing—review & editing. **SK:** Writing—review & editing. **AWK:** Writing—review & editing. **DL:** Writing—review & editing. **LLM:** Writing—review & editing. **EL:** Writing—review & editing. **FL:** Writing—review & editing. **ML:** Writing—review & editing. **JL:** Writing—review & editing. **AL:** Writing—review & editing. **RJM:** Writing—review & editing. **MEM:** Writing—review & editing. **TM:** Writing—review & editing. **CM:** Writing—review & editing. **SLN:** Writing—review & editing. **WGN:** Writing—review & editing. **AN:** Writing—review & editing. **KMO:** Writing—review & editing. **AFO:** Writing—review & editing. **JEO:** Writing—review & editing. **HO:** Writing—review & editing. **NO:** Writing—review & editing. **CMP:** Writing—review & editing. **GP:** Writing—review & editing. **ECP:** Writing—review & editing. **RLP:** Writing—review & editing. **GR:** Writing—review & editing. **HSR:** Writing—review & editing. **KJR:** Writing—review & editing. **DPS:** Writing—review & editing. **CS:** Writing—review & editing. **MJS:** Writing—review & editing. **BS:** Writing—review & editing. **FS:** Writing—review & editing. **CS:** Writing—review & editing. **RJS:** Writing—review & editing. **X-OS:** Writing—review & editing. **AS:** Writing—review & editing. **MCS:** Writing—review & editing. **JJS:** Writing—review & editing. **JS:** Writing—review & editing. **AJS:** Writing—review & editing. **RMT:** Writing—review & editing. **JAT:** Writing—review & editing. **MAT:** Writing—review & editing. **CMV:** Writing—review & editing. **EmvV:** Writing—review & editing. **XW:** Writing—review & editing. **CRW:** Writing—review & editing. **CW:** Writing—review & editing. **WW:** Writing—review & editing. **SJW:** Writing—review & editing. **AW:** Writing—review & editing. **XRY:** Writing—review & editing. **WZ:** Writing—review & editing. **AZ:** Writing—review & editing. **AMD:** Data curation; Funding acquisition; Project administration; Writing—review & editing. **PDPP:** Data curation; Funding acquisition; Project administration; Writing—review & editing. **MKS:** Data curation; Funding acquisition; Project administration; Writing—review & editing. **PK:** Data curation; Funding acquisition; Methodology; Project administration; Writing—original draft; Writing—review & editing. **DFE:** Data curation; Funding acquisition; Project administration; Writing—original draft; Writing—review & editing. **RLM:** Conceptualization; Data curation; Funding acquisition; Project administration; Writing—original draft; Writing—review & editing. **MG-C:** Conceptualization; Data curation; Funding acquisition; Methodology; Project administration; Supervision; Validation; Visualization; Writing—original draft; Writing—review & editing.

## Supplementary Material

djaa056_Supplementary_Data
