# Supplementary material for: Combined Associations of a Polygenic Risk Score and Classical Risk Factors With Breast Cancer Risk
Source: J Natl Cancer Inst. 2020 May 2;113(3):329–37. doi: 10.1093/jnci/djaa056 (PMC7936056; doi:10.1093/jnci/djaa056)
Supplement: djaa056_Supplementary_Data [file djaa056_supplementary_data.pdf]

Supplementary Methods

Study participants

Analyses were conducted using data from 46 studies (16 prospective cohorts, 14 population-based case-control studies and 16 non-population based studies) participating in BCAC (Supplementary Table 1). Participants were excluded if they were male, were of non-European descent, had breast cancer of unknown invasiveness or had in-situ breast tumors. Women with unknown reference age (defined as age at diagnosis for cases and age at interview for controls) and women who had prevalent disease at the time of recruitment were also excluded from the analyses. After implementation of the above exclusion criteria, studies with at least 150 cases and 150 controls having genetic data and information on at least one of the lifestyle risk factor were included in the further analyses. All studies were approved by the relevant ethics committee and written informed consent was acquired from the study participants.

Genetic data

Two custom-made genotyping arrays: iSelect genotyping array (iCOGS) and OncoArray 500K (Oncoarray) were used to genotype the samples. Detailed information about genotyping and imputation can be found elsewhere [1-4]. Briefly, 28,176 cases and 32,209 controls of European ancestry were genotyped by the iCOGS array, containing 211,155 SNPs, and 44,109 cases and 48,145 controls were genotyped by Oncoarray, comprising of 533,000 SNPs, of which 230,000 SNPs served as "GWAS backbone" (Illumina HumanCore).

Epidemiological data

Epidemiological data from different studies was centrally quality controlled and harmonized to a common data dictionary and was derived with respect to a reference date (age at diagnosis for cases and age at interview for controls). The mean (standard deviation) of reference age in the iCOGS data set is 57.5 (11.3) years for cases and 56.8 (11.4) years for controls. In the OncoArray dataset, the mean (standard

deviation) reference age is 59.5 (11.7) years for cases and 57.3 (11.9) years for controls. The following lifestyle risk factors variables were used in the analysis: age at menarche (per 2 years), ever parous (yes or no), ever use of oral contraceptives (yes or no), adult body mass index (BMI) separately for pre- and postmenopausal women (per 5 kg/m^2), adult height (per 5 cm), lifetime alcohol consumption (per 10 g/day), current smoking (yes or no), and family history defined as family history of breast cancer in a first-degree relative (yes or no). Further reproductive variables, including number of full-term pregnancies (1, 2, 3 and ≥4), age at first full-term pregnancy (per 5 years), ever breastfed (yes or no), duration of breastfeeding (per 12 months) were assessed in parous women. Current use of combined estrogen-progesterone menopausal hormonal therapy (MHT) (yes or no) and current use of estrogen-only MHT (yes or no) were analyzed for postmenopausal women. Women were categorized as pre- and postmenopausal based on their self-reported menopausal status. In case of missing menopausal status, reference age (<54 years: premenopausal and ≥54 years: postmenopausal) was used as surrogate to assign menopausal status.

Creation of PRS

Detailed information on creation of PRS is explained in Mavaddat *et al.*[5]. Briefly, using the Breast Cancer Association Consortium data from 69 studies comprising of nearly 94,000 cases and 75,000 controls of European descent, a new 313-SNP PRS was developed. SNPs were sorted and ranked based on their p-value of the associations with overall breast cancer risk. SNPs were then filtered in linkage disequilibrium and correlation such that, uncorrelated SNPs with lowest p-values were taken forward. Two approaches were employed to the remaining SNPs after preliminary filtration: (i) hard thresholding and stepwise forward regression model and (ii) penalized lasso regression method. Effect estimates for all the SNPs chosen by these methods were assessed in a logistic regression model in order to develop a best PRS. For ER-subtype specific PRS, effect estimates were obtained from case-only lasso model, otherwise overall estimates were utilized. The best PRS was further validated in an independent dataset of 10

prospective studies (approximately 11,000 cases and 18,000 controls) and also using data from the UK Biobank cohort (nearly 3,000 breast cancer incident cases).

Individual PRS was derived using the formula

$$PRS = \beta_1 x_1 + \beta_2 x_2 + ... + \beta_k x_k .... + \beta_n x_n$$

where β_k is per-allele log risk ratio (in this case, odds ratio) for breast cancer established with the minor allele of SNP k, x_k is the dosage of the allele for SNP k and n is the total number of SNPs (which is 313 in these analyses). The effect estimates used to construct the PRS_{313} are obtained from Supplementary Table 7 of Mavaddat *et al.*[5]. Subtype-specific PRSs were created by incorporating ER-subtype specific weights.

Overall, the 313-SNP PRS showed evidence of increased risk of overall breast cancer with an odds ratio (OR) of 1.65 (95% CI = 1.59-1.72) per 1 SD for the PRS. This PRS was found to be more predictive for ER-positive breast cancer risk with OR of 1.74 (95% CI = 1.66-1.82) per SD of PRS when compared to ER-negative breast cancer risk (OR = 1.65, 95% CI = 1.59-1.72).

Statistical analysis

Interaction odds ratio (OR) and 95% confidence interval were assessed using unconditional logistic regression and likelihood ratio tests. We also conducted a newly developed case-only method [6] to evaluate the departure from multiplicative model between polygenic risk score (PRS) and lifestyle risk factors. This method takes into account the independence between PRS and risk factors, and has been shown to be more efficient over the logistic regression. The interaction between PRS and risk factors is evaluated using a simple linear regression of the PRS on the risk factors in the sample of cases. To check the independence assumption between the PRS and classical risk factors, we calculated pair-wise Spearman correlations for all variables using unaffected controls (Supplementary Figure 5).

Individual models were fitted for each PRS-risk factor combination for overall and ER-specific breast cancer. The ER-specific PRS was used for interaction analyses of the corresponding ER-specific breast cancer risk. Each model was adjusted for reference age (date at diagnosis for cases and date at interview for controls), study and ten array-specific principal components. An indicator variable for study design was created (prospective cohort/population-based case-control vs. non-population-based studies). To account for potential differential main effects of risk factors by study design (prospective cohort/population-based, non-population based), an interaction term between risk factor and the aforementioned indicator variable was also added to the model, along with main effects. Models assessing current use of menopausal hormonal therapy (MHT) by type (estrogen-progesterone combined (EPT) or estrogen-only therapy (ET)) were further adjusted for former use of any MHT and former use of MHT other than the one being assessed. The association analysis of current smoking was further adjusted for former smoking. Analyses were conducted separately for iCOGS and OncoArray and then results were meta-analyzed using fixed-effect inverse-variance method. Analyses were conducted using SAS 9.4 [7] and R version 3.4.4[8].

Using the population-based studies, we evaluated the goodness-of-fit of a multiplicative model between PRS_{313} and individual risk factors for overall and ER-positive breast cancer risk. Global goodness-of-fit was tested using the Hosmer-Lemeshow (HL) test to compare expected and observed risks by quantiles. Furthermore, goodness-of-fit was tested at the extremes of the distribution (tails) by using the tail-based goodness of fit [9]. Due to relatively small number of cases, goodness of fit was not tested for ER-negative breast cancer risk.

We used the iCARE-BPC3 model [10] to estimate the distribution of lifetime risk of breast cancer for 50-year old White non-Hispanic US women before attaining 80 years. For these calculations, we utilized an individual level reference dataset of risk factors representative of this population [11] as well as breast cancer incidence rates from the US National Cancer Institute-Surveillance, Epidemiology, and End Results Program (NCI-SEER) (2015) and competing mortality rates from the Center for Disease Control

(CDC) WONDER database (2015). We assume that the PRS is independent of the other risk factors, conditional on family history. The genetic risk score accounts for the attenuation of the family history association due to its correlation with the PRS.

For computing the genetic risk score, the log relative risks for all the risk factors except family history and PRS was set to zero. We categorized the population into deciles of the genetic risk score based on the 313-SNP PRS and family history (i.e., presence or absence of breast cancer in first degree relatives) multiplied by the log-relative risk for family history. A new variable was created to record the decile specific average genetic risk score and included as a covariate in the model. The log relative risk for this new variable was set to 1 and the log relative risk for family history was set to 0.

The recently developed Individualized Coherent Absolute Risk Estimator (iCARE) tool was used to perform the above calculations [12]. More specifically the computeAbsoluteRisk() function implemented within the iCARE tool was used. The log relative risk for the risk factors were obtained from Mass, P. et al. [10]. We fitted the multiplicative model presented in this paper and it included an interaction term between BMI and menopausal hormone therapy. Within each category of the genetic risk, we computed the absolute risk in the age range 50-80 years based on a) classical risk factors (i.e., all other risk factors excluding PRS and family history), and b) modifiable classical risk factors (BMI, use of hormonal replacement therapy, smoking status, and alcohol consumption) with the genetic risk score fixed at the category specific average. For computing the absolute risk based on modifiable risk factors, the log relative risk of all the other non-modifiable risk factors are set to zero. More details on the calculation of the absolute risk and the iCARE tool can be found elsewhere [12]. Figure 1a and 1b shows the distribution of this absolute lifetime risk within each category. In calculation of the absolute lifetime risk, we did not include the interaction between family history and PRS, therefore, the absolute lifetime risk may be slightly over-estimated for women with family history and high PRS.

References:

1. Amos CI, Dennis J, Wang Z, *et al.* The OncoArray Consortium: A Network for Understanding the Genetic Architecture of Common Cancers. Cancer Epidemiol Biomarkers Prev 2017;26(1):126-135.
2. Michailidou K, Hall P, Gonzalez-Neira A, *et al.* Large-scale genotyping identifies 41 new loci associated with breast cancer risk. Nat Genet 2013;45(4):353-61, 361e1-2.
3. Michailidou K, Lindstrom S, Dennis J, *et al.* Association analysis identifies 65 new breast cancer risk loci. Nature 2017;551(7678):92-94.
4. Milne RL, Kuchenbaecker KB, Michailidou K, *et al.* Identification of ten variants associated with risk of estrogen-receptor-negative breast cancer. Nat Genet 2017;49(12):1767-1778.
5. Mavaddat N, Michailidou K, Dennis J, *et al.* Polygenic Risk Scores for Prediction of Breast Cancer and Breast Cancer Subtypes. Am J Hum Genet 2019;104(1):21-34.
6. Meisner A, Kundu P, Chatterjee N. Case-Only Analysis of Gene-Environment Interactions Using Polygenic Risk Scores. Am J Epidemiol 2019;188(11):2013-2020.
7. SAS Institute Inc. Cary NC. In.
8. R Core Team. R: A language and environment for statistical computing. Vienna, Austria: R Foundation for Statistical Computing; 2017. In.
9. Song M, Kraft P, Joshi AD, *et al.* Testing calibration of risk models at extremes of disease risk. Biostatistics 2015;16(1):143-54.
10. Maas P, Barrdahl M, Joshi AD, *et al.* Breast Cancer Risk From Modifiable and Nonmodifiable Risk Factors Among White Women in the United States. JAMA Oncol 2016;2(10):1295-1302.
11. Choudhury PP, Wilcox AN, Brook MN, *et al.* Comparative validation of breast cancer risk prediction models and projections for future risk stratification. J Natl Cancer Inst 2019; 10.1093/jnci/djz113.
12. Choudhury PP, Maas P, Wilcox A, *et al.* iCARE: R package to build, validate and apply absolute risk models. 2018; 10.1101/079954 %J bioRxiv:079954.

Supplementary Figures

Supplementary figure 1: Associations of main effect of the PRS (by percentiles) on overall and estrogen receptor (ER)-subtype breast cancer risk in this dataset

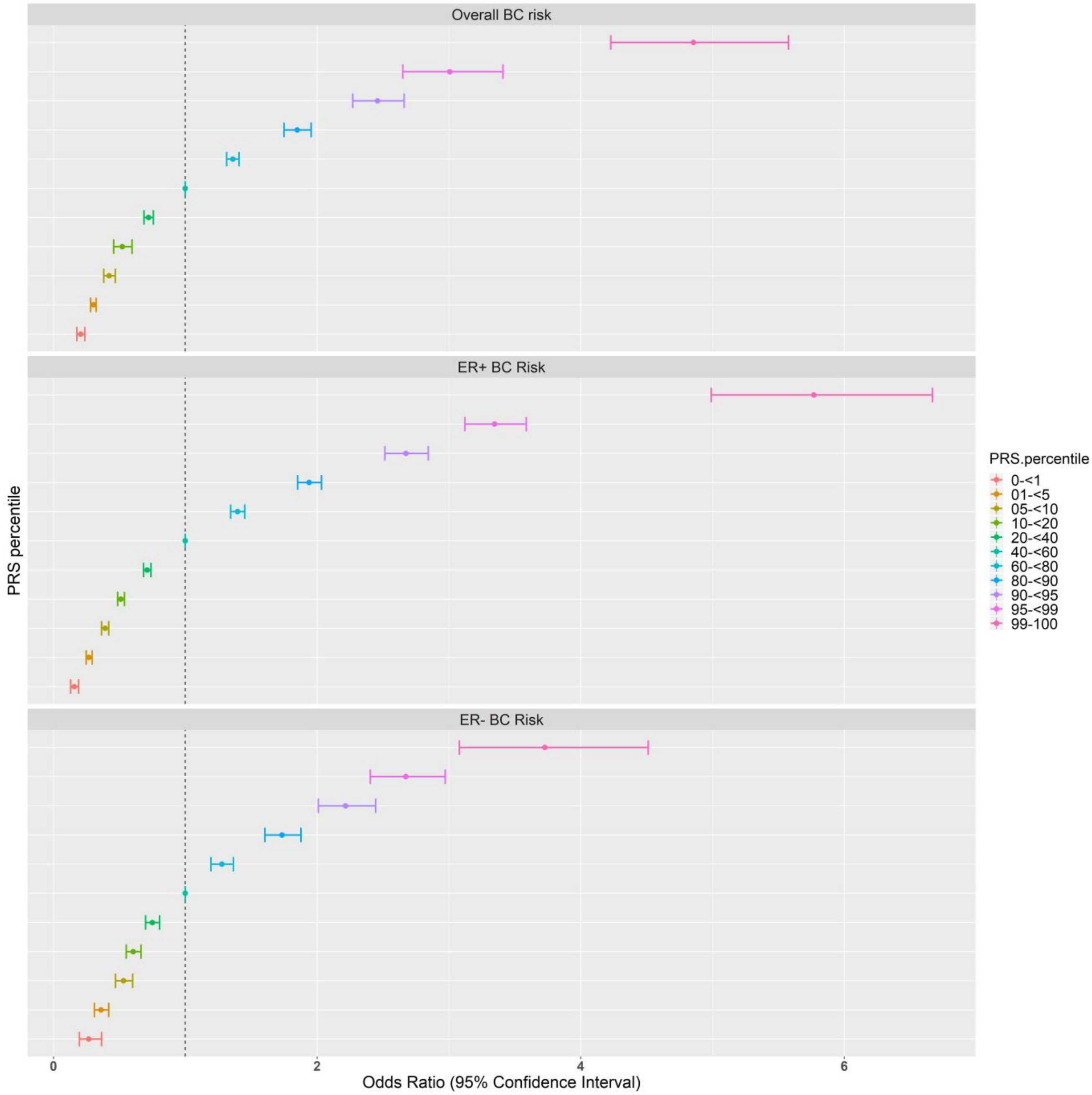

BC: Breast cancer, PRS: Polygenic risk score, ER: estrogen receptor, ER+: Estrogen receptor positive, ER-: Estrogen receptor negative.

X-axis shows the odds ratio and y-axis shows the PRS percentiles. The legend on the right side shows the corresponding color scheme of PRS percentiles.

Supplementary Figure 2: Odd ratios and 95% confidence intervals for classical risk factors by percentiles of the 313-SNP polygenic risk score for overall breast cancer risk

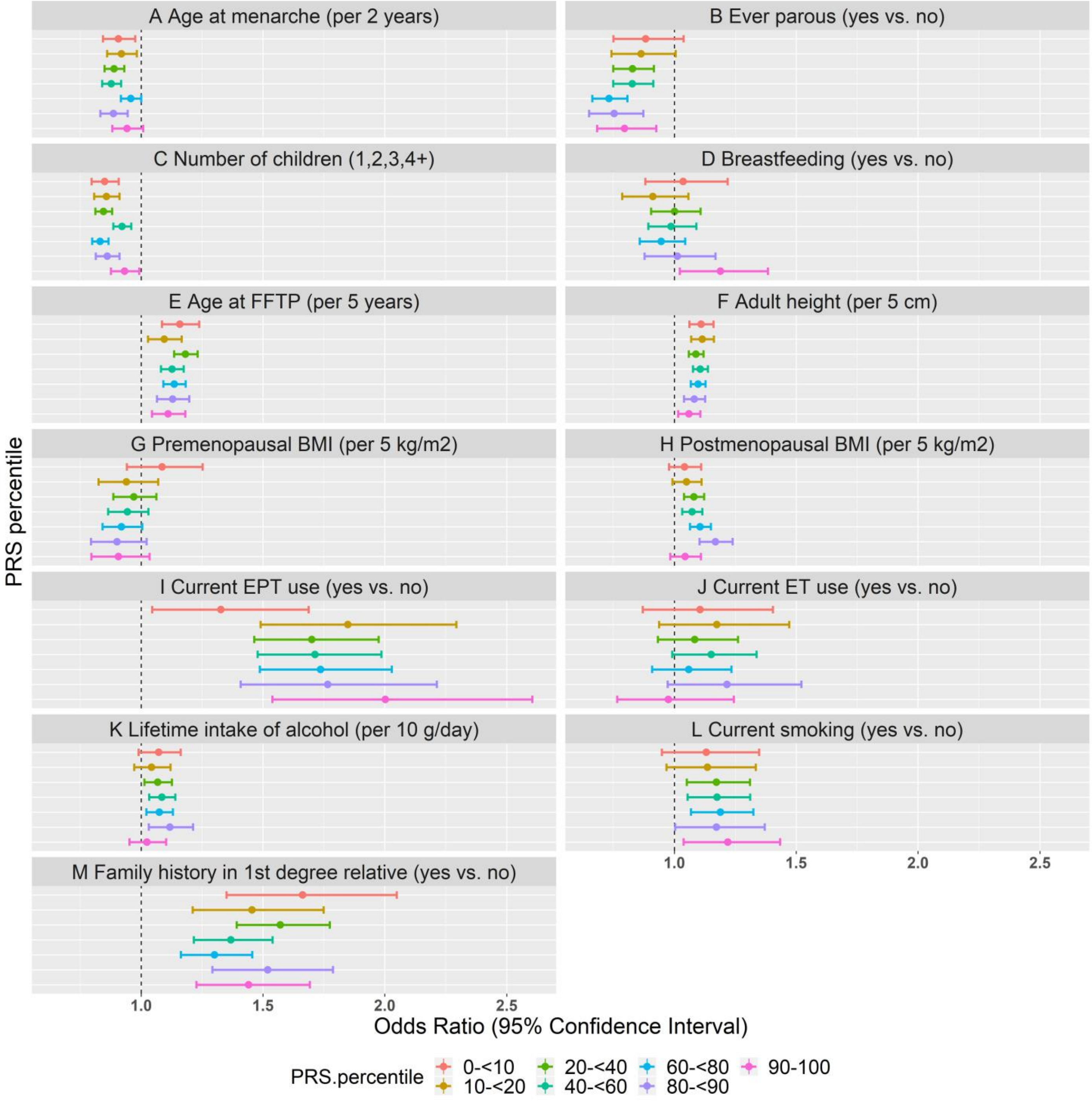

PRS: Polygenic risk score, FFTP: First full-term pregnancy, BMI: Body mass index, EPT: Estrogen-progesterone therapy, ET: Estrogen-only therapy

X-axis shows the odds ratio and y-axis shows the PRS percentiles. The legend on the right side shows the corresponding color scheme of PRS percentiles.

Supplementary Figure 3: Odd ratios and 95% confidence intervals for classical risk factors by percentiles of the 313-SNP polygenic risk score for ER-positive breast cancer risk

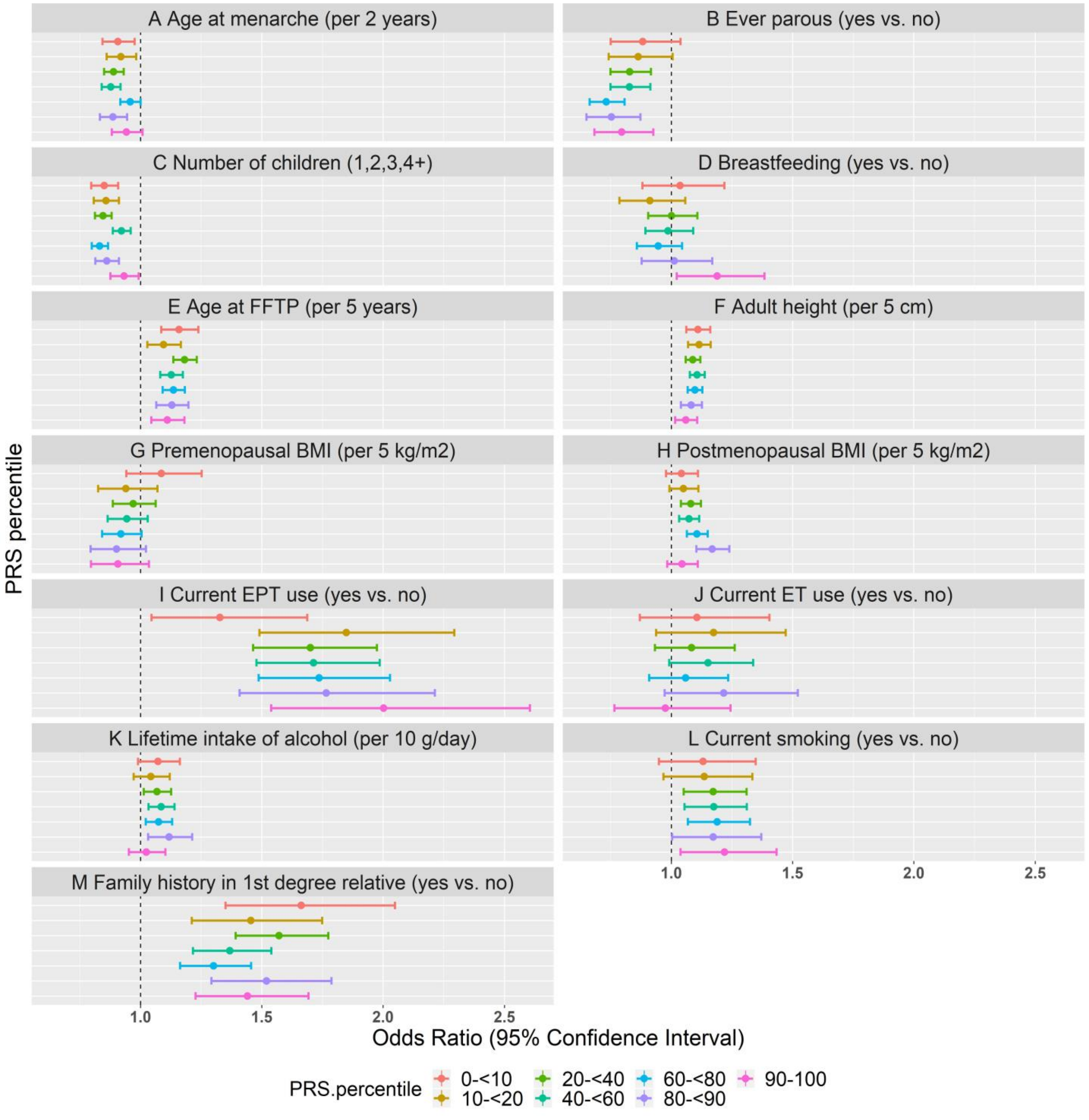

PRS: Polygenic risk score, FFTP: First full-term pregnancy, BMI: Body mass index, EPT: Estrogen-progesterone therapy, ET: Estrogen-only therapy

X-axis shows the odds ratio and y-axis shows the PRS percentiles. The legend on the right side shows the corresponding color scheme of PRS percentiles.

Supplementary Figure 4: Odd ratios and 95% confidence intervals for classical risk factors by percentiles of the 313-SNP polygenic risk score for ER-negative breast cancer risk

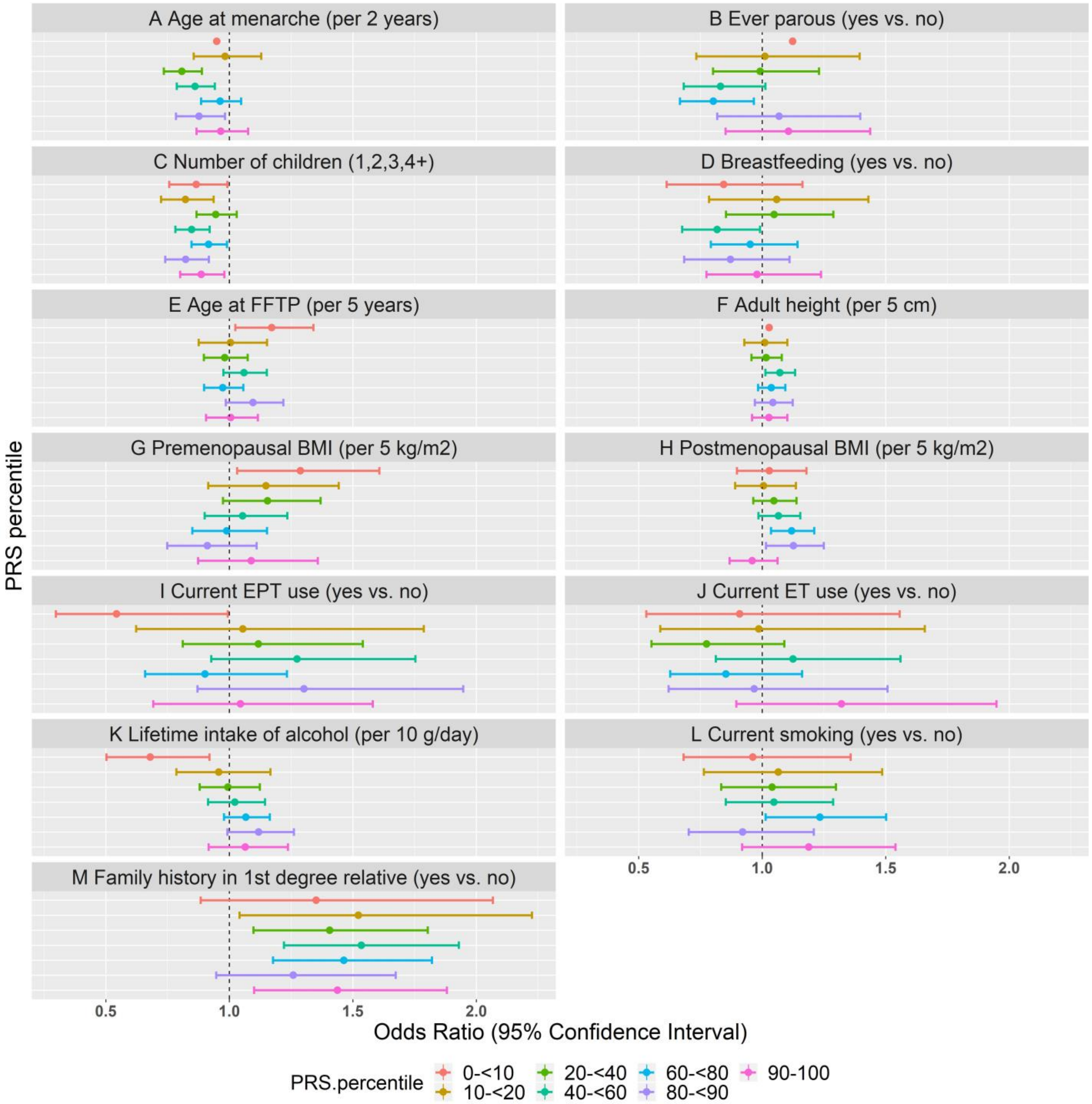

PRS: Polygenic risk score, FFTP: First full-term pregnancy, BMI: Body mass index, EPT: Estrogen-progesterone therapy, ET: Estrogen-only therapy

X-axis shows the odds ratio and y-axis shows the PRS percentiles. The legend on the right side shows the corresponding color scheme of PRS percentiles.

Supplementary Figure 5: Heatmap of Spearman pairwise correlation between PRS_{313} (overall and ER-subtype) and all classical risk factors (high positive correlation: red, high negative correlation: blue) using controls.

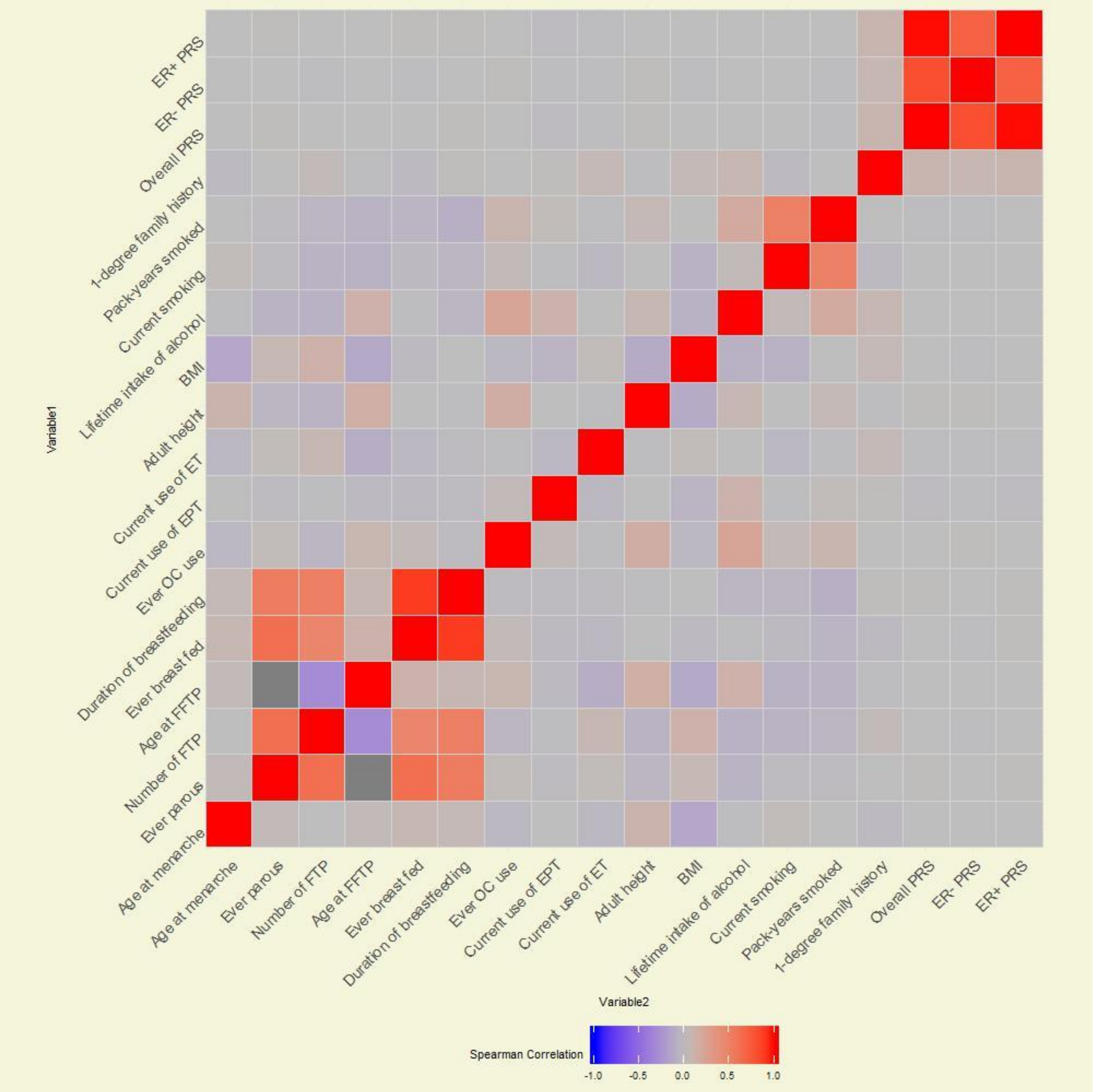

ER+: Estrogen receptor positive, ER-: Estrogen receptor negative, PRS: Polygenic risk score, BMI: Body mass index, ET: Estrogen-only menopausal hormonal therapy, EPT: Combined estrogen-progesterone therapy, OC: Oral contraceptives, FFTP: First full-term pregnancy, FTP: Full-term pregnancies, 1-degree family history: Family history in first degree relative.

Supplementary Tables

Supplementary Table 1: List of participating studies with number of total cases and controls

Study name	*Study acronym*	*Country*	*Study Design*[1]	*Cases*	*Controls*
Australian Breast Cancer Family Study	ABCFS	Australia	Population-based case-control study	1317	738
Amsterdam Breast Cancer Study	ABCS	Netherlands	Non population-based study	442	1376
Australian Breast Cancer Tissue Bank	ABCTB	Australia	Non population-based study	947	375
Agricultural Health Study	AHS	USA	Prospective cohort study	513	1137
Bavarian Breast Cancer Cases and Controls	BBCC	Germany	Non population-based study	809	706
Breast Cancer Employment and Environment Study	BCEES	Australia	Population-based case-control study	783	834
Breast Cancer in Northern Israel Study	BCINIS	Israel	Population-based case-control study	1315	724
Breast Oncology Galicia Network	BREOGAN	Spain	Non population-based study	1265	725
Canadian Breast Cancer Study	CBCS	Canada	Population-based case-control study	568	817
CECILE Breast Cancer Study	CECILE	France	Population-based case-control study	910	1002
Copenhagen General Population Study	CGPS	Denmark	Non population-based study	4064	5241
Spanish National Cancer Centre Breast Cancer Study	CNIO-BCS	Spain	Non population-based study	746	829
Cancer Prevention Study-II Nutrition Cohort	CPSII	USA	Prospective cohort study	2546	3323
California Teachers Study	CTS	USA	Prospective cohort study	1156	610
European Prospective Investigation Into Cancer and Nutrition	EPIC	France, Germany, Greece, Italy, Spain, The Netherlands, and UK	Prospective cohort study	3436	3597
ESTHER Breast Cancer Study	ESTHER	Germany	Population-based case-control study	476	505
Gene Environment Interaction and Breast Cancer in Germany	GENICA	Germany	Population-based case-control study	912	710
Genetic Epidemiology Study of Breast Cancer by Age 50	GESBC	Germany	Population-based case-control study	316	181
Karolinska Mammography Project for Risk Prediction of Breast Cancer - Cohort Study	KARMA	Sweden	Prospective cohort study	1415	6026
Kathleen Cuningham Foundation Consortium for research into Familial Breast Cancer/Australian Ovarian Cancer Study	KCONFAB/AOCS	Australia and New Zealand	Non population-based study	251	896
Leuven Multidisciplinary Breast Centre	LMBC	Belgium	Non population-based study	3003	1821

Mammary Carcinoma Risk Factor Investigation	MARIE	Germany	Population-based case-control study	1643	2065
Mayo Clinic Breast Cancer Study	MCBCS	USA	Non population-based study	2062	2041
Melbourne Collaborative Cohort Study	MCCS	Australia	Prospective cohort study	1002	1206
Multiethnic Cohort	MEC	USA	Prospective cohort study	668	724
Melanoma Inquiry of Southern Sweden	MISS	Sweden	Prospective cohort study	599	1529
Mayo Mammography Health Study	MMHS	USA	Prospective cohort study	276	1635
Nashville Breast Health Study	NBHS	USA	Population-based case-control study	482	652
Northern California Breast Cancer Family Registry	NC-BCFR	USA	Non population-based study	696	150
North Carolina Breast Cancer Study	NCBCS	USA	Population-based case-control study	2074	1006
Nurses' Health Study	NHS	USA	Prospective cohort study	1103	1804
Nurses' Health Study 2	NHS2	USA	Prospective cohort study	1112	1905
Ontario Familial Breast Cancer Registry	OFBCR	Canada	Non population-based study	1934	728
NCI Polish Breast Cancer Study	PBCS	Poland	Population-based case-control study	1768	2082
Karolinska Mammography Project for Risk Prediction of Breast Cancer - Case-Control Study	PKARMA	Sweden	Non population-based study	3115	5464
The Prostate, Lung, Colorectal and Ovarian Cancer Screening Trial	PLCO	USA	Prospective cohort study	1822	2595
Predicting the Risk Of Cancer At Screening Study	PROCAS	UK	Population-based case-control study	342	1656
Singapore and Sweden Breast Cancer Study	SASBAC	Sweden	Population-based case-control study	1129	1373
Sheffield Breast Cancer Study	SBCS	UK	Non population-based study	594	848
Study of Epidemiology and Risk factors in Cancer Heredity	SEARCH	UK	Non population-based study	12571	8889
The Sister Study	SISTER	USA	Prospective cohort study	1501	1562
Swedish Mammography Cohort	SMC	Sweden	Prospective cohort study	1349	661
UCI Breast Cancer Study	UCIBCS	USA	Non population-based study	427	258
UK Breakthrough Generations Study	UKBGS	UK	Prospective cohort study	1047	1032
US Radiologic Technologists Study	USRT	USA	Non population-based study	848	1699
Women's Health Initiative Observational Study	WHI	USA	Prospective cohort study	4930	4617
Total				72284	80354

[1]Population-based design was defined as recruiting a random sample of all cases occurring in a geographically defined population during a specified period of time, and recruiting controls that were a random sample of the same source population as cases during the same period of time. Non-population-based design was defined as not strictly population-based (e.g. due to oversampling of selected participant groups for genotyping) or hospital-based.

Supplementary Table 2: Characteristics of the study population by study design.

Characteristics	Population-based studies				Non population-based studies			
	Cases N (%)	Controls N (%)	Cases Mean (S.D.)	Controls Mean (S.D.)	Cases N (%)	Controls N (%)	Cases Mean (S.D.)	Controls Mean (S.D.)
**Reference age**	38510	48308	61.03 (11.27)	58.66 (10.71)	33774	32046	56.10 (11.38)	54.80 (12.58)
**ER status**								
Positive	27830 (72.27)				22385 (66.28)			
Negative	5783 (15.02)				5113 (15.14)			
Missing	4897 (12.72)				6276 (18.58)			
**Menopausal status**								
Premenopausal	9045 (23.49)	12047 (24.94)			12556 (37.18)	13424 (41.89)		
Postmenopausal	29465 (76.51)	36261 (75.06)			21218 (62.82)	18622 (58.11)		
**Family history in a first-degree relative**								
Yes	7226 (18.76)	6784 (14.04)			5396 (15.98)	2060 (6.43)		
No	19564 (50.80)	28860 (59.74)			19764 (58.52)	15895 (49.60)		
Missing	11720 (30.43)	12664 (26.22)			8614 (25.50)	14091 (43.97)		
**Reproductive risk factors**								
**Age at menarche (years)**	36893	46855	12.86 (1.53)	12.96 (1.56)	22415	19439	12.99 (1.57)	12.99 (1.55)
**Ever parous**								
Yes	32025 (83.16)	41555 (86.02)			20442 (60.53)	23398 (73.01)		
No	5217 (13.55)	5618 (11.63)			3933 (11.65)	4127 (12.88)		
Missing	1268 (3.29)	1135 (2.35)			9399 (27.83)	4521 (14.11)		
**Number of full-term pregnancies**[a]								
1	5572 (17.40)	6182 (14.88)			3912 (19.14)	4151 (17.74)		
2	13004 (40.61)	17091 (41.13)			9811 (47.99)	11263 (48.14)		
3	7735 (24.15)	10427 (25.09)			4511 (22.07)	5140 (21.97)		
≥4	5323 (16.62)	7652 (18.41)			2048 (10.02)	2146 (9.17)		
Missing	391 (1.22)	203 (0.49)			160 (0.78)	698 (2.98)		
**Ever breastfed**[a]								

Yes	17358 (54.20)	19953 (48.02)			11298 (55.27)	9543 (40.79)		
No	6155 (19.22)	6557 (15.78)			3167 (15.49)	2409 (10.30)		
Missing	8512 (26.58)	15045 (36.21)			5977 (29.24)	11446 (48.92)		
**Duration of breast feeding[a] (months)**	20737	22183	7.83 (10.39)	8.30 (10.77)	9201	5555	6.99 (10.22)	7.32 (10.67)
**Age at FFTP[a] (years)**	30412	39987	24.92 (4.65)	24.67 (4.54)	17883	16192	25.09 (5.05)	25.48 (4.82)
**Anthropometric risk factors**								
**Adult height (cm)**	35767	46506	163.58 (6.50)	163.62 (6.49)	23642	18359	164.13 (6.78)	164.54 (6.88)
**Premenopausal BMI[b] (kg/m^2)**	8509	11510	24.85 (4.78)	25.26 (5.12)	8467	9464	25.28 (4.95)	24.98 (4.78)
**Postmenopausal BMI[c] (kg/m^2)**	28069	35112	26.52 (5.29)	26.05 (4.98)	14877	15508	26.45 (4.99)	26.30 (4.85)
**Hormonal risk factors**								
**Ever use of oral contraceptives**								
Yes	19632 (50.98)	26311 (54.47)			11018 (32.62)	12356 (38.56)		
No	15750 (40.90)	18441 (38.17)			5080 (15.04)	3419 (10.67)		
Missing	3128 (8.12)	3556 (7.36)			17676 (52.34)	16271 (50.77)		
**Current use of EPT[c]**								
Yes	3490 (11.84)	2758 (7.61)			258 (1.22)	174 (0.93)		
No	13525 (45.90)	16757 (46.21)			3962 (18.67)	3406 (18.29)		
Missing	12450 (42.25)	16746 (46.18)			16998 (80.11)	15042 (80.78)		
**Current use of ET[c]**								
Yes	2736 (9.29)	3236 (8.92)			185 (0.87)	240 (1.29)		
No	14072 (47.76)	16180 (44.62)			3929 (18.52)	3282 (17.62)		
Missing	12657 (42.96)	16845 (46.45)			17104 (80.61)	15100 (81.09)		
**Lifestyle risk factors**								
**Lifetime intake of alcohol (g/day)**	15829	18723	6.55 (12.57)	5.79 (10.33)	1461	1376	10.84 (14.81)	33.60 (63.31)
**Current smoking**								
Yes	4762 (12.37)	5630 (11.65)			2505 (7.42)	14681 (45.81)		
No	28975 (75.24)	37592 (77.82)			11965 (35.43)	15214 (47.48)		
Missing	4773 (12.39)	5086 (10.53)			19304 (57.16)	14681 (45.81)		

**Pack-years smoked[d]**	11607	15660	17.82 (18.27)	15.64 (16.44)	2969	3980	18.31 (17.63)	16.03 (15.58)

This table shows the number of cases and controls for each risk factor after all exclusions except for the exclusion of 150 cases and 150 controls for the variable of interest. This exclusion was conducted individually for each risk factor at the time of fitting logistic regression models. For continuous variables mean and standard deviation are reported, whereas, for categorical variables numbers and percentage are reported.

N: Number; %: Percentage; S.D.: Standard deviation; ER: Estrogen receptor; FFTP: First full-term pregnancy; BMI: Body mass index; EPT: Combined estrogen-progesterone menopausal hormonal therapy; ET: Estrogen-only menopausal hormonal therapy

[a] Among parous women, [b] Among premenopausal women, [c] Among postmenopausal women, [d] Among women who were ever smokers

Supplementary Table 3: Associations of epidemiological risk factors for overall and ER-specific subtype breast cancer risk in population-based and cohort studies

Environmental risk factor	*Overall breast cancer risk OR (95% CI)*	*ER-positive breast cancer risk OR (95% CI)*	*ER-negative breast cancer risk OR (95% CI)*
Univariate models[a]			
Age at menarche (per 2 years)	0.91 (0.89-0.92)	0.91 (0.89-0.93)	0.89 (0.85-0.93)
Ever parous (yes/no)	0.81 (0.77-0.84)	0.78 (0.74-0.81)	0.94 (0.85-1.04)
Number of full-term pregnancies (1,2,3,≥4)[1]	0.87 (0.85-0.88)	0.86 (0.84-0.87)	0.90 (0.86-0.94)
Age at first full-term pregnancy (per 5 years)[1]	1.14 (1.12-1.16)	1.17 (1.14-1.19)	1.02 (0.97-1.06)
Ever breastfed (yes/no)[1]	0.91 (0.88-0.95)	0.92 (0.88-0.96)	0.96 (0.88-1.03)
Duration of breastfeeding (per 12 months)[1]	0.96 (0.93-0.98)	0.95 (0.93-0.98)	0.98 (0.94-1.03)
Adult height (per 5 cm)	1.09 (1.08-1.10)	1.10 (1.09-1.12)	1.03 (1.00-1.05)
Premenopausal BMI (per 5 kg/m^2)	0.95 (0.92-0.98)	0.92 (0.89-0.95)	1.07 (0.98-1.16)
Postmenopausal BMI (per 5 kg/m^2)	1.07 (1.05-1.09)	1.07 (1.05-1.09)	1.05 (1.00-1.11)
Ever use of oral contraceptives (yes/no)	1.22 (1.18-1.26)	1.24 (1.20-1.29)	1.14 (1.05-1.23)
Current use of EPT (yes/no)[2,3]	1.75 (1.65-1.87)	1.93 (1.81-2.06)	1.11 (0.92-1.34)
Current use of ET (yes/no)[2,3]	1.10 (1.03-1.17)	1.11 (1.03-1.19)	1.35 (1.11-1.64)
Lifetime intake of alcohol (per 10 g/day)	1.07 (1.05-1.10)	1.09 (1.07-1.11)	1.03 (0.98-1.08)
Current smoking (yes/no)[4]	1.18 (1.13-1.24)	1.18 (1.12-1.25)	1.06 (0.96-1.18)
Pack years smoked (per 10 pack-years)[5]	1.02 (1.00-1.04)	1.02 (1.00-1.04)	1.00 (0.95-1.04)
Family history (yes/no)	1.56 (1.49-1.64)	1.54 (1.46-1.62)	1.53 (1.39-1.68)
Multivariate model 1[b]			
Age at menarche (per 2 years)	0.89 (0.86-0.94)	0.90 (0.86-0.95)	0.85 (0.79-0.93)
Number of full-term pregnancies (per 1 unit)	0.89 (0.86-0.93)	0.88 (0.85-0.92)	0.89 (0.82-0.95)
Age at first full-term pregnancy (per 5 years)	1.07 (1.02-1.12)	1.08 (1.03-1.14)	0.96 (0.88-1.04)
Ever breastfed (yes/no)	0.97 (0.89-1.05)	0.98 (0.89-1.07)	0.96 (0.82-1.12)
Adult height (per 5 cm)	1.05 (1.01-1.08)	1.07 (1.03-1.10)	0.99 (0.94-1.05)
BMI (<18.5 kg/m^2)	1.28 (0.92-1.76)	1.35 (0.94-1.92)	1.13 (0.66-1.96)
BMI (25-30 kg/m^2)	1.04 (0.93-1.16)	1.00 (0.88-1.13)	1.12 (0.92-1.36)
BMI (≥30 kg/m^2)	1.14 (0.98-1.33)	1.02 (0.86-1.22)	1.03 (0.76-1.38)
Current use of MHT (yes/no)	1.30 (1.15-1.46)	1.39 (1.22-1.58)	1.00 (0.80-1.24)
Interaction between current use of MHT and BMI (<18.5 kg/m^2)	0.61 (0.33-1.13)	0.55 (0.28-1.07)	1.12 (0.39-3.23)
Interaction between current use of MHT and BMI (25-30 kg/m^2)	0.91 (0.74-1.12)	0.93 (0.74-1.16)	0.82 (0.54-1.26)
Interaction between current use of MHT and BMI (≥30 kg/m^2)	0.98 (0.72-1.34)	1.02 (0.72-1.43)	0.69 (0.33-1.42)

Lifetime intake of alcohol (per 10 g/day)	1.02 (0.99-1.06)	1.02 (0.99-1.06)	1.04 (0.99-1.10)
Current smoking (yes/no)	1.28 (1.16-1.42)	1.36 (1.22-1.52)	1.01 (0.85-1.20)
Family history (yes/no)	1.75 (1.57-1.94)	1.73 (1.54-1.94)	1.72 (1.42-2.08)
Multivariate model 2[c]			
BMI (<18.5 kg/m^2)	1.10 (0.87-1.39)	1.22 (0.94-1.58)	1.04 (0.67-1.61)
BMI (25-30 kg/m^2)	0.99 (0.92-1.07)	0.96 (0.88-1.04)	1.04 (0.90-1.21)
BMI (≥30 kg/m^2)	1.10 (1.00-1.22)	1.05 (0.94-1.18)	1.13 (0.91-1.39)
Current use of MHT (yes/no)	1.45 (1.34-1.57)	1.58 (1.45-1.72)	1.09 (0.92-1.28)
Interaction between current use of MHT and BMI (<18.5 kg/m^2)	0.75 (0.49-1.16)	0.63 (0.39-1.01)	0.99 (0.43-2.31)
Interaction between current use of MHT and BMI (25-30 kg/m^2)	0.88 (0.77-1.00)	0.90 (0.78-1.04)	0.94 (0.70-1.27)
Interaction between current use of MHT and BMI (≥30 kg/m^2)	0.86 (0.72-1.03)	0.88 (0.72-1.07)	0.60 (0.37-2.61)
Lifetime intake of alcohol (per 10 g/day)	1.05 (1.03-1.08)	1.07 (1.04-1.09)	1.02 (0.98-1.07)
Current smoking (yes/no)	1.28 (1.19-1.38)	1.31 (1.21-1.42)	1.12 (0.97-1.28)

BMI: Body mass index, EPT: Estrogen-Progesterone menopausal hormonal therapy, ET: Estrogen-only menopausal hormonal therapy; MHT: Menopausal hormonal therapy

[a]All OR estimates are based on a model with single risk factor analyses adjusted for reference age and study

[b] Model includes all classical risk factors: age at menarche, age at first full-term pregnancy, number of children, ever breastfed, height, BMI (18.5-<25 kg/m^2: reference category), current MHT use, current smoking, and lifetime alcohol consumption. This model is adjusted for reference age, study, menopausal status, former smoking, former use of menopausal hormonal therapy, interaction between BMI and current MHT use, and interaction between BMI and former MHT use

[c] Model includes modifiable risk factors: BMI (18.5-<25 kg/m^2: reference category), current MHT use, current smoking and lifetime alcohol consumption. This model is adjusted for reference age, study, menopausal status, former smoking, former use of menopausal hormonal therapy, interaction between BMI and current MHT use, and interaction between BMI and former MHT use.

[1] among parous women

[2] among postmenopausal women

[3] Additionally, models were adjusted for former use of menopausal hormonal therapy and use of any other menopausal hormonal therapy preparations

[4] Additionally, model was adjusted for former smoking

[5] among ever smokers

Supplementary table 4: Goodness of fit test p-values for overall breast cancer and estrogen receptor (ER) positive breast cancer, based on population-based studies

Single risk factor models with 313-SNP PRS[1]	**Overall breast cancer risk**			**ER-positive breast cancer risk**		
	Cases/Controls	*Tail-based goodness-of-fit test*	*Global goodness-of-fit test*	*Cases/Controls*	*Tail-based goodness-of-fit test*	*Global goodness-of-fit test*
Age at menarche (per 2 years)	36893/46855	0.75	0.47	26331/46855	0.41	0.67
Ever parous (yes/no)	37242/47173	0.68	0.28	26938/47173	0.34	0.62
Number of children (1, 2, 3, ≥4)[2]	31634/41352	0.40	0.19	22860/41352	0.29	0.08
Age at first full-term pregnancy (per 5 years)[2]	30412/39987	0.87	0.42	22009/39987	0.78	0.40
Breastfeeding (yes/no)[2]	23513/26510	0.23	0.32	16616/26510	0.45	0.76
Duration of breastfeeding (per 12 months)	20737/22183	0.09	0.08	14615/22183	0.16	0.73
Adult height (per 5 cm)	35767/46506	0.51	0.60	25764/46506	0.18	0.44
Premenopausal BMI (per 5kg/m^2)[3]	8509/11510	0.51	0.56	5615/11510	0.74	0.31
Postmenopausal BMI (per 5kg/m^2)[4]	28069/35112	0.31	0.65	20884/35112	0.09	0.24
Ever use of oral contraceptives (yes/no)	35382/44752	0.30	0.38	25417/44752	0.13	0.53
Current use of EP therapy (yes/no)[4,5]	17015/19515	0.43	0.31	12777/19515	0.55	0.78
Current use of E-only therapy (yes/no)[4,5]	16808/19416	0.48	0.26	12614/19416	0.63	0.79
Alcohol consumption (per 10g/day)	15829/18723	0.40	0.11	11304/18723	0.07	0.44
Current smoking (yes/no)[6]	33737/43222	0.51	0.82	24124/43222	0.20	0.68
Pack-years (per 10 pack-years)	11607/15660	0.71	0.95	8373/15660	0.46	0.80

[1]always adjusted for study; [2]in parous women only; [3] in premenopausal women; [4] in postmenopausal women; [5] adjusted for former use of MHT and use of any other MHT than the preparation of interest; [6] adjusted for former smoking.
